# Building Trust for Smart Connected Devices: The Challenges and Pitfalls of TrustZone

**DOI:** 10.3390/s21020520

**Published:** 2021-01-13

**Authors:** Nikolaos Koutroumpouchos, Christoforos Ntantogian, Christos Xenakis

**Affiliations:** 1 Department of Digital Systems, University of Piraeus, 18534 Piraeus, Greece; nikoskoutr@ssl-unipi.gr; 2 Department of Informatics, Ionian University, 49100 Corfu, Greece; dadoyan@ionio.gr

**Keywords:** TrustZone, Trusted Execution Environments, vulnerabilities, exploitation, side channel attacks, IoT

## Abstract

TrustZone-based Trusted Execution Environments (TEEs) have been utilized extensively for the implementation of security-oriented solutions for several smart intra and inter-connected devices. Although TEEs have been promoted as the starting point for establishing a device root of trust, a number of published attacks against the most broadly utilized TEE implementations request a second view on their security. The aim of this research is to provide an analytical and educational exploration of TrustZone-based TEE vulnerabilities with the goal of pinpointing design and implementation flaws. To this end, we provide a taxonomy of TrustZone attacks, analyze them, and more importantly derive a set of critical observations regarding their nature. We perform a critical appraisal of the vulnerabilities to shed light on their underlying causes and we deduce that their manifestation is the joint effect of several parameters that lead to this situation. The most important ones are the closed implementations, the lack of security mechanisms, the shared resource architecture, and the absence of tools to audit trusted applications. Finally, given the severity of the identified issues, we propose possible improvements that could be adopted by TEE implementers to remedy and improve the security posture of TrustZone and future research directions.

## 1. Introduction

Due to the emergence and adaptation of smart connected devices as a platform for interaction with an ever-expanding number of digital services and systems, security has emerged as a major design goal for these smart connected devices. The ability to connect, manage, and control a device from anywhere and at any time leads such devices to generate, process, and exchange vast amounts of security-critical and privacy-sensitive data, turning them into attractive cyberattack targets. The strong intra and inter-connectivity of smart connected devices requires a holistic, end-to-end security approach, addressing security and privacy risks at all levels. 

Security by isolation is a well-established strategy for achieving security goals such as data confidentiality, integrity, and availability. Several software-based approaches such as microkernels, sandboxes, and virtualizations have been used, but these methods fail in providing the desired security level [1,2]. Smart connected devices must be fortified with new security-oriented technologies that guarantee security from the outset.

Among existing security-oriented technologies, TrustZone is gaining particular attention due to the massive presence of ARM processors in the embedded market [3]. TrustZone technology is an implementation of a Trusted Execution Environment (TEE), which is a hardware and software based isolated, secure container of state and data that functions in parallel to the main OS of the system. In TrustZone-based TEEs, security-sensitive applications and sensitive data can be offloaded with the purpose of enhancing their protection and assurance of their integrity and confidentiality. The need for a TEE is derived by the realization that the standard operating system of the platform is incapable of safeguarding the integrity of the applications and their data. The design goal of TEE is to be able to host a wide variety of security-sensitive applications and to be able to protect -mainly by hardware driven isolation- their own internal state’s integrity as also that of the serving applications, from malicious modification.

ARM TrustZone have been researched and proposed as providers of security sensitive solutions in various application domains of smart connected devices including sensors [4], Industry 4.0 and IoTs [5], Vehicular communications [6], drones [7] and wearables [8]. However, an ever-expanding number of discovered vulnerabilities and developed attacks against the most widely used TEE implementations, poses a question on the ability of current designs to achieve the standing of the most secure element on the platform, as they are been heavily promoted of from the side of both platform designers and integrators [9,10,11,12,13,14].

This paper illuminates this reality and projects the requirement for a more extensive security awareness from the perspective of their design and implementation as well. More specifically, first we provide essential information about the TrustZone security extensions, TEE implementations and types of services they host. Next, we elaborate on the discovered vulnerabilities and related attacks using a taxonomy that classifies them into three main categories: (a) software, (b) architectural, and (c) side channel attacks. The presentation of the attacks is comprehensive providing a set of critical observations that carry out the most important outcomes of each attack for pedagogical purposes. We pinpoint that an attacker with the ability to execute code in the context of the secure world can fully compromise the system, while also have access to secrets stored within the secure world. Furthermore, an attacker that has kernel rights within TrustZone can manipulate resources from other trusted applications. We also provide generic attack paths to visually demonstrate all possible vectors that attackers can exploit to compromise the security of TrustZone. Next, we perform a critical appraisal of the discovered vulnerabilities to shed light on the roots of these subtle security flaws. Finally, we provide important security guidelines and solutions both for application developers and TEE implementors to remedy and improve the overall security posture of TrustZone. In summary, this work provides the following contributions:

1. Proposes a taxonomy of the currently known public attacks against TEE implementations and conducts an in-depth analysis of them.
2. Provides high-level attack paths to describe how vulnerabilities in different architectural components can result in partial or complete takeover of the TrustZone.
3. Provides insights into the possible reasons behind the security weaknesses.
4. Proposes guidelines that developers and security practitioners should follow in order to enhance the security status of TrustZone and suggests future research directions.
5. Draws attention in a very relevant area of research since trusted computing is one of the key factors for applying security and trust in next generation IoT and sensor networks.

There are various works that investigate TEEs and their provided security guarantees, compare their capabilities with other related technologies and analyze their shortcomings [15,16,17,18,19,20,21]. In [15] researchers developed a theoretical model for TEEs, analyze its core components and propose a more precise definition of it. Although they are referencing some of the public attacks on TEEs, they do not analyze the impact of them nor the reasons that may lead to their existence. In [16] Arfaoui et al. provide a view of the various existing TEE technologies according to GlobalPlatform standards. [17] Presents a survey of trust computing in mobile devices. Moreover, in [18] there is a comparative security analysis of the TrustZone and the SGX technologies while in [19,20,21] the researchers provide a comprehensive analysis of the TrustZone technology in general with no emphasis on the attacks against it. The focus of the above papers is different from our approach, as none of the three exposes the degree of vulnerability and the importance of the various TrustZone-based TEE solutions used in market deployed devices, while they fail to pinpoint common design flaws that lead to these vulnerabilities. Finally, concurrent to our work, Cerdeira et al. [22] presented and provided a comprehensive taxonomy of TrustZone-based TEE vulnerabilities. The results of this paper are complementary to our research and analysis.

The rest of this paper unfolds as follows: Section 2 provides background information for the ARM TrustZone technology to facilitate better understating of the presented notions. Section 3 provides an analytical and educational analysis of TrustZone-based TEE vulnerabilities and related attacks. Section 4 highlights important observations related to the roots of the discovered vulnerabilities and discusses possible solutions to the identified issues as well as future research directions. Finally, Section 5 includes the conclusions.

## 2. Background

### 2.1. ARM TrustZone and Trusted Execution Environments Architecture

The use of a secure processor environment can be traced back to 2003 in Texas Instruments OMAP processors [23] which were deployed on Nokia phones (particularly OMAP 161x and 73x processors). Later in 2015, Texas Instruments branded the technology as M-Shield [24]. The ARM TrustZone hardware security extensions were introduced into Cortex-A processors in 2004 [25]. The first standardization attempt of TEEs was described publicly by Open Mobile Terminal Platform (OMTP) in its specification for Advanced Trusted Environment in 2009. One year later in 2010, the center of TEE standardization became another industry forum, GlobalPlatform, which published its first TEE standard, TEE client API 1.0 that defines the communication between trusted applications which are executed in TEE, and applications executed by the main operating system.

The ARM TrustZone has been introduced for the Cortex-A processors (which is the focus of this paper), while more recently it has been extended to the new generation of ARM microcontrollers called Cortex-M designed for low-cost and energy efficient devices. It is worth mentioning that there are similarities and important differences in the implementation of TrustZone between the two processor families [26]. The most deployed TrustZone-based TEEs in the majority of devices currently in the market are Trustsonic’s Kinibi (or <t-base/mobicore) [27] and Qualcomm’s QSEE [28]. Other industrial implementations consist of Huawei’s HiSilicon TEE [29], SecuriTEE of Solacia [30], Microsoft-Nokia’s implementation named ObC [31], SierraTEE [32] and Google’s Trusty TEE [33]. All of the above with the exception of ObC (which was discontinued in 2013) and Trusty are closed source systems with little public knowledge available about the internals of their design and their code base. Except of the industrial solutions, academic implementations exist also. Genode TEE of Genode Labs [34], OP-TEE [35], OpenTEE of Intel Collaborative Research Institute for Secure Computing [36], Andix OS of TU Graz University of Technology [15], ARMithril of North Carolina State University [15], SafeG of Nagoya University [37], ViMoExpress defined by Electronics and Telecommunication Research Institute [38], and TLR of Microsoft Research [39] are the most commonly referenced academic TEEs.

As a feature, TrustZone can been seen as a special kind of hardware-supported virtualization of CPU state, memory, interrupt signals and I/O data, with the purpose of isolation. This virtualization technology enables each physical CPU core to provide an abstraction of two virtual ones, orthogonally dividing the process state in two logical realms, or worlds as they are named: The normal world of the Rich Execution Environment (REE) and the secure world of TEE. In this way it allows a TEE to run in parallel and isolated from the main REE where the standard OS (Android, iOS, etc.) and standard user applications exist. In the TEE, a security-oriented OS is operating and serves a number of security sensitive applications called trustlets or Trusted Applications (TAs), providing through them services to the normal world OS and to the platform. Each side of the platform has a different view of memory, interrupts and I/O hardware with the secure world being the most privileged. A third element, the monitor mode, acts as a gatekeeper between the two worlds and manages switching between the two and the transitions and transfers of state in a secure way [15].

World switching between the two execution environments is facilitated via the Secure Monitor Call (SMC) instruction. In normal operation the non-secure operating system processes tasks in the standard way. When a non-privileged REE process requires services of a trusted application running in the secure execution environment, it requests an execution state transfer to the privileged non-secure kernel. The non-secure kernel through a device driver, by use of the SMC instruction, calls the secure monitor to undertake the switch to the secure world [40,41]. Apart from the software SMC call, a subset of hardware exception mechanisms containing the IRQs, FIQs, external data abort and external prefetch abort are able to modify the current state of execution and cause a world switch.

Furthermore, the ARMv8 architecture follows an exception model that is divided in four different privilege levels that follows the classic software segregation between application and operating system/kernel privileges [42]. In this way, it separates the system into relatively privileged abstractions. Each privilege level is denoted by its associated Exception Level (EL0, EL1, EL2, EL3) with EL0 being the lowest privileged while EL3 the highest. This model propagates throughout the normal and the secure world, affecting their applications and operating systems. More specifically, the unprivileged level of execution EL0 is where the user space applications reside like trusted and normal world applications, while the normal world and secure world operating systems and their kernels belong to EL1. The various hypervisors used for virtualization of the normal world or the secure world EL1 operating systems [43,44] reside in EL2 and can essentially allow the parallel execution of multiple EL1 operating systems but they cannot manage or manipulate the secure state of the system; that is, a transition between the secure and the normal world. This privilege is only allowed to the final and most privileged EL3 where the secure monitor (trusted firmware) is the only entity allowed in this exception level.

**Figure 1:**
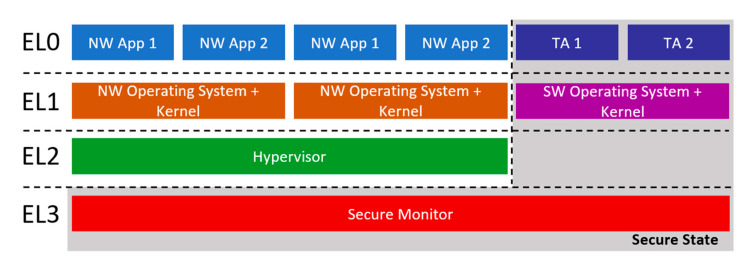
ARMv8 exception level architecture.

The secure monitor typically provides a basic set of functionalities [45] that include: a Power State Coordination Interface (PSCI) for coordinated power management [46], a Trusted Board Boot Requirements CLIENT (TBBR-CLIENT) for the trusted boot process and the isolation between the normal and secure world [47], an SMC Calling Convention for SMC handling [48], a System Control and Management Interface (SCMI) for coordinated system control and management tasks [49] and a Software Delegated Exception Interface (SDEI) for registering and servicing system events [50]. Finally, EL0-EL2 are separated in two states, secure and non-secure which are usually denoted with NS.EL [0–2] and S.EL [0–2] and they are used to provide a distinction between the normal and the secure world; the EL3 is always in the secure state. The secure state of the processor is managed by the SCR_EL3.NS bit. A depiction of this exception level architecture can be found in Figure 1.

**Figure 2:**
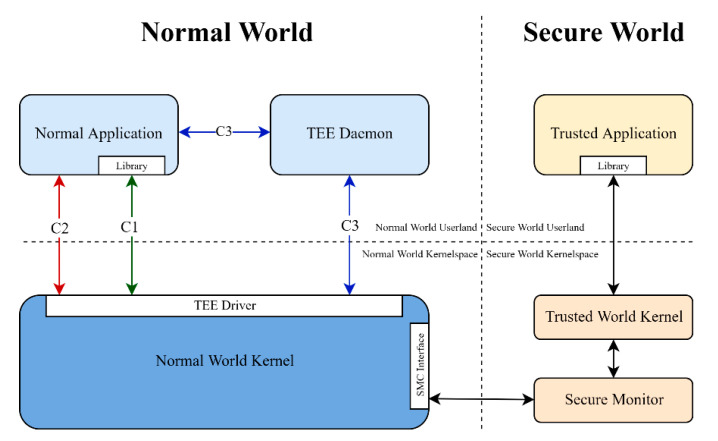
Communication channels between the normal and the secure world.

In Figure 2, there is a demonstration of how the two worlds are divided between them and their respective kernels. Each world includes its user-space and kernel-space for internal operations with the secure monitor being a part of the secure world. The communication between the two worlds can be implementation-dependent with three options being the most common which can be seen in Figure 2: (C1), a library is provided which directly communicates with the underlying TEE driver, (C2) the user application directly communicates with the TEE driver and (C3) there is a running daemon responsible for managing and forwarding requests to the TEE driver. Regardless of the chosen normal world implementation, the TEE driver will undergo any required procedures in order to create an SMC system call, which in turn hand over the execution to the secure monitor. Then, the secure monitor will assess the SMC and pass the execution to the trusted world kernel that will finally call the appropriate trusted application. It should be noted here that the two worlds, in order to communicate properly, have methods for sharing data either through small variables or shared memory objects.

The sharing of memory between the secure and the normal world is implemented with two different SMC calls: the fast call and the yield call (defined in the ARM trusted firmware reference implementation as SMC_TYPE_FAST and SMC_TYPE_YIELD) [51]. The fast call provides a fast register-based information exchange, where only up to four variables can be used to transfer limited data between the two worlds. These variables can be used by both the normal and the secure world to transfer function parameters, operational results, and any other required data. On the other hand, the yield call allocates a memory location of the normal world to be shared with the secure world, this call is useful for scenarios where large amounts of data need to be transferred or when there is a need for synchronous trusted applications like DRM protected video streaming.

Additionally, TrustZone facilitates two components for memory management and protection, the TrustZone Memory Adapter (TZMA) and the TrustZone Address Space Controller (TZASC), respectively. These components implement protection schemes through static partitioning of the on-chip memory (TZMA) and through dynamic partitioning of the off-chip memory. The memory management units are configured by early bootloaders to only allow specific execution environments to access specific memory locations. If the normal world environment attempts to read or write the memory that is configured in a memory controller for the TrustZone kernel, the CPU will abort and, depending on configuration, reboot the device due to the violation.

**Figure 3:**
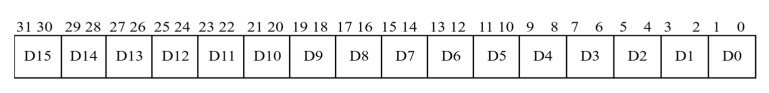
The DACR register, each memory region is assigned 2 Bits.

On top of these TrustZone features, ARM also implements an access permission scheme that defines whether memory access is allowed or not, based on special permission characteristics assigned to each memory page. For instance, a read and execute-only page (which usually are code pages) cannot be modified, while other memory regions that will hold dynamic data should be assigned writeable permissions too. This is enforced through an access control mechanism that utilizes the Domain Access Control Register or DACR. The MMU checks if the bits corresponding to the given memory region in the DACR register (of the ARM specification) are set; If so, the access is allowed. The DACR is a 32-bit register, which specifies the access properties of 16 memory domains (2 bits for each domain, see Figure 3). The possible values are [52]:

- 00: Access is denied. Any access generates a domain fault.
- 01: Access is granted depending on the permissions of the actor. (Read/Write/Execute)
- 10: Reserved. By default, access is denied.
- 11: Access is unconditionally granted.

Thus, if the value of the DACR register bits of the corresponding memory domain are set to 11, the MMU will enable access to any memory address of that domain for both writing and reading without generating any faults.

For bus management, two interfaces exist: the advanced extensible Interface (AXI) and the Advanced Peripheral Bus (APB), both acting under the AMBA3 [53] specifications. The first implements the bus interface for the main SoC level system and the second is a low bandwidth simple peripheral bus interface, which is attached to the system using an AXI-to-APB bridge. The primary interface has the capability –through an extended signaling system and the insertion of a 33rd flag bit named NS-bit- to separate peripherals based on a secure or normal world division schema. The APB bus has no capabilities of this nature and the responsibility of managing security must be handled by the aforementioned intermediate bridge interconnect. Additionally, the system can have both secure and non-secure hardware peripherals that respective secure and non-secure device drivers control, from each mode independently through the provided buses [40].

### 2.2. GlobalPlatform Specification & Protection Profile

Regarding standardization activities, GlobalPlatform is the main organization creating and publishing specifications for the TEE technology. Initially focusing on TEE system architecture specifications and on standardization of the interface between the TEE and the REE (client API), a specification for the interface between the TEE applications and the Secure OS (internal API) was later provided. They have also published a TEE protection profile that targets threats to the TEE assets that arise during the end-usage phase and can be achieved by software means [26]. Most ARM TrustZone-based TEEs which are available in commodity devices, strictly conform to these standards.

In the protection profile specification of GlobalPlatform, multiple attributes and requirements are defined that provide the scope of the security protection that a TEE should provide. That is what security guarantees are mandatory for TEE implementors. This document presents: (i) the target of evaluation for the main security features and intended uses of a TEE device, (ii) the conformity of the document to common criteria, (iii) the security problem definition, (iv) the security objectives and (v) the security requirements. Furthermore, in the appendix of the document there is a description of potential attacks to TEEs with varying profiles and scenarios that also include hardware attacks (side channel attacks and fault injection attacks) which the TEE should be able to defend against to some extent. 

The part of the document that we focused on is the security objectives which will allow us to assess each attack by investigating which security objective is violated through it. In the following list we shortly describe each mandatory TEE objective and in how many attacks we found a violation of it. In Section 3.4 which is analyzed after the attacks presentation in Section 3.4 we will showcase exactly which of those objectives is violated by each attack.

- O.CA_TA_IDENTIFICATION: Protection for the identity of each trusted application from being used from other trusted applications. Also, assurance for the distinction of normal world applications and trusted applications. (Violations: 0)
- O.KEYS_USAGE: Enforcement of defined usage restrictions to cryptographic keys. (Violations: 0)
- O.TEE_ID: Statistical uniqueness and protection of the TEE identifier generated by the TEE. (Violations: 0)
- O.INITIALISATION: Secure boot process that protects the integrity of the TEE initialization code and data, the authenticity of the TEE firmware, the binding of the TEE firmware to the hardware and the protection against TEE firmware downgrade attacks. (Violations: 4)
- O.INSTANCE_TIME: Provisioning of monotonic trusted application instance time. (Violations: 0)
- O.OPERATION: Correct operation of its security functions by (i) protecting itself against bugs and violation of good practices from the normal world and also the trusted applications, (ii) control access to its services by the REE and trusted applications, (iii) have a secure state fallback upon failure detection without exposure of sensitive data. (Violations: 26)
- O.RNG: Cryptographically secure random number generation. (Violations: 0)
- O.RUNTIME_CONFIDENTIALITY: Confidentiality of TEE and trusted application runtime data and keys. (Violations: 12)
- O.RUNTIME_INTEGRITY: Integrity of volatile memory stored TEE firmware and runtime data, trusted application code and runtime data/keys. (Violations: 16)
- O.TA_AUTHENTICITY: Verification of authenticity of trusted applications’ binary code. (Violations: 1)
- O.TA_ISOLATION: Isolation between trusted applications. (Violations: 8)
- O.TEE_DATA_PROTECTION: Protection of TEE persistent data. (Violations: 2)
- O.TEE_ISOLATION: Isolation of TEE context from the trusted applications and the normal world. (Violations: 10)
- O.TRUSTED_STORAGE: Protection of the TEE-provided permanent trusted storage that includes both keys and data and its proper functionality. (Violations: 12)

### 2.3. TEE Capabilities and Types of Trusted Applications

Inside the TEE, Platform/OEM engineers in collaboration with third parties, can implement several security oriented and security sensitive services utilizing its assurance and secure storage capabilities, aiming for integrity and confidentiality of their assets. In current implementations a centralized provision model is used, with the developer of the corresponding TEE acting as the central authority that will allow or deny a service to be installed on its TEE. The capabilities a TEE offers can be generalized into the below categories:

- Isolated Execution
- Secure Storage
- Device Identification
- Device Authentication
- Device Attestation
- Platform Integrity

Utilizing the above core capabilities, TrustZone TEEs can provide a wide range of functionalities such as: verification of kernel integrity, access to secure credential generation, secure storage (Android Keystore, dm-verity), secure element emulation for secure mobile payments, enforcement of corporate policies, implementation and verification of secure boot, content protection, digital rights management solutions (PlayReady, Widevine, etc.) and device integrity attestation in the scope of IoT and ARM Cortex-M; although its performance could be optimized for low powered devices with novel attestation schemes [54].

From the scope of what is targeted by each solution, we can divide them into three categories: services that enhance platform and normal world security, services that provide functionalities to the user and enhance user security, and services towards third parties. In the first category, solutions like Samsung’s TIMA (a proprietary solution limited to Samsung devices) [55], secure boot [56] and Sprobes [57] provide real-time protection, integrity verification and introspection mechanisms for the security enhancement of the normal world kernel/OS. Secure key storage, software TPM [58,59,60], trusted peripherals and sensors [4] are other paradigms of solutions that can fit this category. Towards user functionality and security enhancement, a wide variety of implemented solutions exist. Online prepaid mobile payments [61,62], online transactions confirmations [63], ticketing services [64], cloud storage access authentication [65,66], two factor authentication [67,68] are some examples. In the third category, solutions like [69,70] provide protection for media content and smart contract execution respectively.

### 2.4. Implementation Details of QSEE

In this section, we further elaborate on QSEE architecture, due to the fact that the majority of the devices and documented attacks are based on this solution. QSEE is the TrustZone-based TEE implementation that Qualcomm has installed in its SoCs. Like all TEEs, QSEE includes user-mode applications in normal world that may require communicating with trusted applications of the secure world in the TEE. For instance, the Android process in charge of handling cryptographic keys named “KeyStore”, needs to be able to communicate with a special trusted application (named “KeyMaster”) which provides secure management of cryptographic keys using the capabilities offered by TrustZone (e.g., secure storage, isolation, etc.). However, user-mode applications cannot perform SMC calls to enter the secure world, since they require kernel-space privileges. Qualcomm TEE implementations solve this problem by using a Linux kernel driver (Figure 2, C2), called “qseecom”, which enables user-space processes to perform a wide range of TrustZone-related operations, such as loading trusted applications into the secure environment and communicating with loaded trusted applications. The qseecom driver provides an ioctl system call interface to perform SMC calls from the kernel-space in place of the normal world application that needs the TEE functionality. The interface between the normal and secure world through the qseecom driver is named Secure Channel Manager (SCM). This channel is the largest attack surface to the TEE, as it provides one of the few handles that the outside world can use to communicate with the trusted world.

Because of the security concern outlined above, the access to qseecom driver is restricted to the minimal set of processes that require it. For example, in the implementation discussed in [71], only four processes of the normal world are able to access “qseecom”:

- SurfaceFlinger (running with “system” user-ID): an Android system service, responsible for compositing all the application and system surfaces into a single buffer that is finally to be displayed by display controller.
- DrmServer (running with “drm” user-ID): which is responsible for digital rights management.
- MediaServer (running with “media” user-ID): that handles the media services of Android.
- KeyStore (running with “keystore” user-ID): a service that is responsible for storing, generating, and handling cryptographic keys in Android.

The aforementioned four processes and any process that can access the QSEE should not be vulnerable, otherwise, if an attacker manages to exploit any of them, then he can directly access any trusted application bypassing the Linux kernel in the process (depending on the vulnerability found). To make things worse, trusted applications are not written in memory safe languages (typically they are written in the C language) which can allow attackers to propagate the exploitation to the secure world. As we will see, QSEE lacks or has weak implementations of common security mechanisms such as ASLR, stack cookies and guard pages [9].

Qualcomm implements the TrustZone fast and yield TEE communication commands (presented in Section 2.1 and Section 2.4) by defining two distinct calls named scm_call() (yield) and scm_call_atomic [1,2,3,4]() (fast). The scm_call() function is used to create and share a common memory region to be used for the communication between the two worlds and it populates a structure which defines the size of the shared buffer, the headers section of the buffer, the offset of the data to be sent and the offset of the receiving data. On the other hand, the scm_call_atomic [1,2,3,4]() functions are used to initiate a communication that uses 1 to 4 variables (depending on the name of the function call) and is mainly used for one-off sessions where the data exchange between the two worlds is relatively small.

Due to the fact that the memory allocated by the scm_call() function might not be within writable or readable regions; it is first validated by the TrustZone kernel before it is used by the trusted application. The validation is done in order to make sure the physical address is within an allowed range, and is not for example, within the TrustZone kernel memory range because as we analyze below, any modification to TrustZone kernel pages is prohibited. The first line of defense is the ARM DACR mechanism analyzed in Section 2.1. We will see that an attacker can potentially send pointers to the secure world bypassing their sanitization.

TrustZone enabled Qualcomm SoCs on top of DACR implement another hardware-based memory access control mechanism in newer devices. More specifically, some predefined memory regions are flagged by the manufacturer as write protected, a policy which is enforced by a hardware memory protection unit (MPU). In Qualcomm, these units are called XPUs and are configured by the OEM during the manufacturing process. XPUs do not only prevent access from normal word to secure world but it also controls the access rights on any physical memory region defined by the manufacturer. For example, the TrustZone kernel code segments are mapped with read-only access permissions by utilizing the XPU mechanism and are verified during the secure boot process. This means that once TrustZone kernel code is loaded into memory, it theoretically cannot (and should not) be subject to any change.

**Figure 4:**
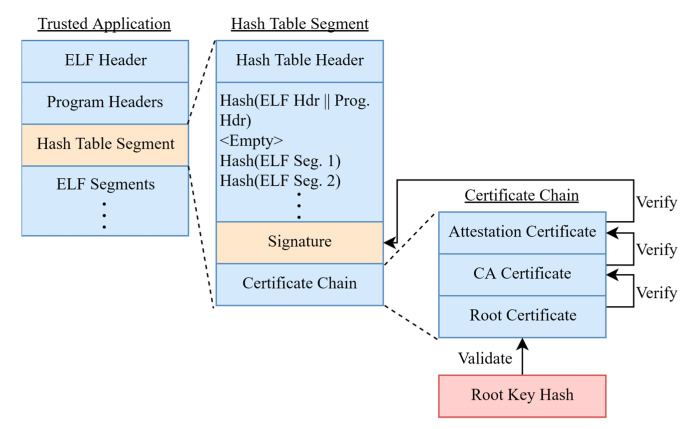
Architecture of a QSEE trusted application.

Another important implementation detail is how loading of trusted applications and their revocation is performed during the secure boot process of Qualcomm. Qualcomm’s signed images are regular ELF files which are supplemented by a single special “Hash Table Segment”. This segment includes three distinct components: the SHA-256 digest of each ELF segment, a signature blob, and a certificate chain. The signature is verified over the concatenated blob of SHA-256 hashes, using the public key of the last certificate in the certificate chain (Attestation Cert). Moreover, the root certificate is hashed and validated against a “Root Key Hash” which is stored in the device’s ROM or fused into one-time-programmable memory on the SoC. This way a chain of trust is created, beginning from a hardware-bound key, each certificate in the chain is validated with the binary signature finally being validated by the last certificate (see Figure 4). Furthermore, it appears that Qualcomm has elected to add unique OU fields to the signature of the binary, denoting several attributes relating to specific attributes that will improve the security of the images loaded. These attributes are:

- **SW_ID:** The software version that this signature belongs to. This acts as a version access control system where its value is compared to an internal eFuse (registers which can only be incremented). If the eFuse comparison indicates that the binary to be loaded is older than the last loaded binary, then it is rejected.
- **HW_ID:** Attribute to bind the binary to a specific r device family, model, and OEM.
- **DEBUG:** Indicates whether the binary should have enabled or disabled debugging features.
- **OEM_ID:** The ID of the OEM from HW_ID.
- **SW_SIZE:** The size of the signed data. (Hash Table Segment)
- **MODEL_ID:** The ID of the model from HW_ID.
- **SHA256/SHA1:** The hashing scheme used for signature verification.

Finally, it is important to mention that Qualcomm TEEs and TEEs in general are considered high privileged entities. While the secure world can secure its own memory, there is no inherent mechanism for the secure world to guarantee the safety of operations on the normal world memory. This lack of information about the non-secure world from within the secure world places a great deal of responsibility on the untrusted OS to sanitize any inputs, especially pointers, that are passed into the secure world. The trusted world can manipulate any memory address of the normal world that is not protected by the DACR or XPUs without taking into consideration the province of that address. Therefore, a compromised TEE can easily be used to attack the normal world kernel even if the kernel had no vulnerabilities to begin with (simply by directly modifying the kernel’s code from the “Secure World”).

### 2.5. Hardware Attacks

The ARM SoCs include a CPU cache that is utilized to improve data and instruction fetch times. This cache is shared by both the secure and the non-secure world while it incorporates the TrustZone NS-bit mechanism to ensure that each world is separated and has proper access rights to its resources. More specifically each cache entry is tagged with the NS-bit so that if a non-secure access attempt is made against a secure entry a cache miss will occur [72]. This might lead to the belief that the cache is safe to be used by the trusted world but recent attacks [73,74,75,76] have shown that there is a major problem in this shared cache design.

The main problem here is the fact that the cache is shared between the two worlds and besides the fact that it is protected by TrustZone, an attacker might find indirect ways to infer secrets from the trusted domain. This kind of attack is called a side channel attack and its basic characteristic is that the attacking entity tries to measure specific parameters of the victim process that will ultimately provide the attacker with enough data to recover the information targeted in the first place.

In the case of caches an attacker can measure two basic things, the execution time of the victim process and which cache lines where accessed. The basic assumption for a cache-based side channel attack is that the attacker needs to be able to manipulate the cache in some way so as to achieve a known state of the cache before it lets the victim process to take control. There are three basic techniques for cache-based side channel attacks:

- Evict + Time [77]: Initially the execution time of the victim program is measured without any changes. Afterwards specific cache sets are evicted that were previously used by the victim program so that in the next execution of the program the time will potentially change. These changes in execution time are correlated to the changes that are made to the cache so that useful information can be extracted which can lead up to secret cryptographic key exposure in the case of AES.
- Prime + Probe [77]: The entire cache is first filled (primed) with known and controlled data by the attacking process. Then the victim process executes while the attacker closely monitors how the initial known state of the cache is changing. Depending on the cache changes that he detects he can obtain information of the victim operation and the nature of its behavior. The main benefit of this technique is that it does not require a shared memory map between the victim and the attacker, making it optimal for targeting the trusted world.
- Flush(Evict) + Reload [74]: For this attack to work the attacker and the victim need to share an identical read-only memory object. The attacker first flushes (or evicts if flush is not available) any cache lines that map to the shared memory object and allows the victim process to run. Afterwards the attacker measures the time it takes to run its own instance of the program so that he can tell what parts of the shared memory object where already loaded in the cache. This is a powerful attack because the attacker can learn exactly what data were loaded in the cache.
- Flush + Flush [78]: This attack is the reverse version of the flush + reload attack. The attacker initially flushes the cache lines that map to the shared object (like before) and then he lets the victim program to run. In the next step, instead of running the program, he flushes the cache once again while measuring the flushing time. Depending on what cache lines were loaded during the victim execution the timing of the flushing operation will change and the attacker will be able to infer information about the victim process. The main benefit of this attack is that it is stealthier from the previous ones because it does not cause any cache misses during its execution something that many attack detection mechanisms seek to identify a cache-based attack.

Aside from the cache-based side-channel attacks we have seen this far, there is another major category of side-channel attacks named fault attacks. The target of these attacks is to induce faults during specific operations so that the process is manipulated in producing results that expose secret information. The main application of fault attacks can be found in differential fault analysis, which is a technique that specifically targets cryptographic algorithms, but it is also used in attacking programs in order to force them down specific control branches during their execution. Faults can be induced with techniques that utilize electromagnetic radiation [76], operation voltage change [79] and operation frequency change [10]. This list is not complete, but its aim is to show the diversity of the possible attack paths and the difficulty of the defense against them.

A recent popular fault attack is the rowhammer attack that targeted the RAM of the victim device [80]. The RAM is composed of capacitors that can store an electric charge, if a capacitor has a charge then it represents a logical one, if not it represents a zero, this way the RAM can store binary data. In recent RAM implementations (after DDR3) these capacitors have shrunken down so that more of them can be installed in the same die, something that has made each capacitor susceptible to interference from neighboring capacitor electromagnetic radiation. This is the attack model of rowhammer, capacitors neighboring to the victim RAM line that contains data to be faulted, are rapidly activated (recharged so that they do not lose their charge) so that the target row will be altered (faulted).

## 3. Attacks on Current Implementations

In this section, we elaborate on the discovered vulnerabilities and exploits. We will focus mainly on QSEE, since it the most widely deployed TEE implementation [81] and most vulnerabilities have been discovered for this platform. An exception is the analysis of a set of discovered attacks for the Kinibi TEE implementation (see Section 3.1.2) and attacks on the TrustedCore of Huawei (see Section 3.1.3). Moreover, the presented attacks are solely based on Cortex-A TrustZone, as Cortex-M is still in its early stages of growth and it is not widely deployed.

**Figure 5:**
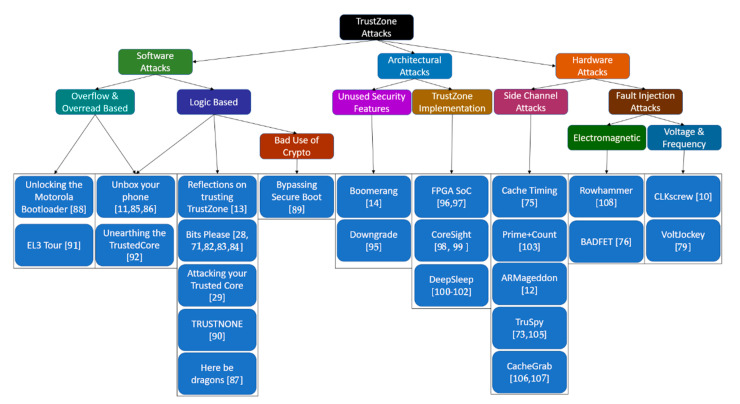
TrustZone attack taxonomy.

We have developed a taxonomy that aims to provide a better understanding of the analyzed TrustZone attacks. The taxonomy firstly differentiates each attack based on three generic categories, software attacks, architectural attacks, and hardware attacks. Software attacks are focused on exploiting the software stack of the implementation, including the different operating systems and the applications that run on the system. The architectural attacks are based on the general architecture of the system that is comprised of both the design of the underlying hardware and the software that runs on the system. The main difference that architectural attacks have is that they exploit a fundamental design flaw of the system and not a specific bug found by accident. Finally, hardware attacks utilize any possible side channel as well as fault injection to transfer information from the trusted world to the normal world, these channels include execution times, cache timing attacks and power usage. For each one of the above three generic categories, the proposed taxonomy makes the following subcategorization (see Figure 5):

(1) **Software Attacks**
  (1.1) Buffer Overflow & Overread Based: Attacks that exploit common memory attacks (buffer overflows/overreads)
  (1.2) Logic Based: Attacks that exploit logical bugs such as faulty comparison branches, incompatible variable types etc.
  (1.3) Bad Use of Crypto: Insufficient strength of used cryptography such as usage of digest functions for authenticated secure integrity verification.
(2) **Architectural Attacks**
  (2.1) Unused Security Features: Unused security features such as downgrade prevention functions or pointer sanitization utilities.
  (2.2) Underlying Architecture: Exploitation of underlying architectural features of the TrustZone implementation such as debugging functions, device sleep features and interconnects.
(3) **Hardware Attacks**
  (3.1) Side Channel Attacks: Exfiltration of data from the device by passively monitoring hardware components (volatile memory) of the device and inferring data from them.
  (3.2) Fault Injection Attacks: Exfiltration of data from the device by monitoring hardware components while actively forcing faulty operations to execute.
    (3.2.1) Electromagnetic: Fault injection attacks that use electromagnetic means to induce faults.
    (3.2.2) Voltage & Frequency: Fault injection attacks that use voltage and/or the CPU frequency to induce faults.

### 3.1. Software Attacks

#### 3.1.1. Bits Please (Logic-Based)

##### Privilege Escalation to TrustZone

**Figure 6:**
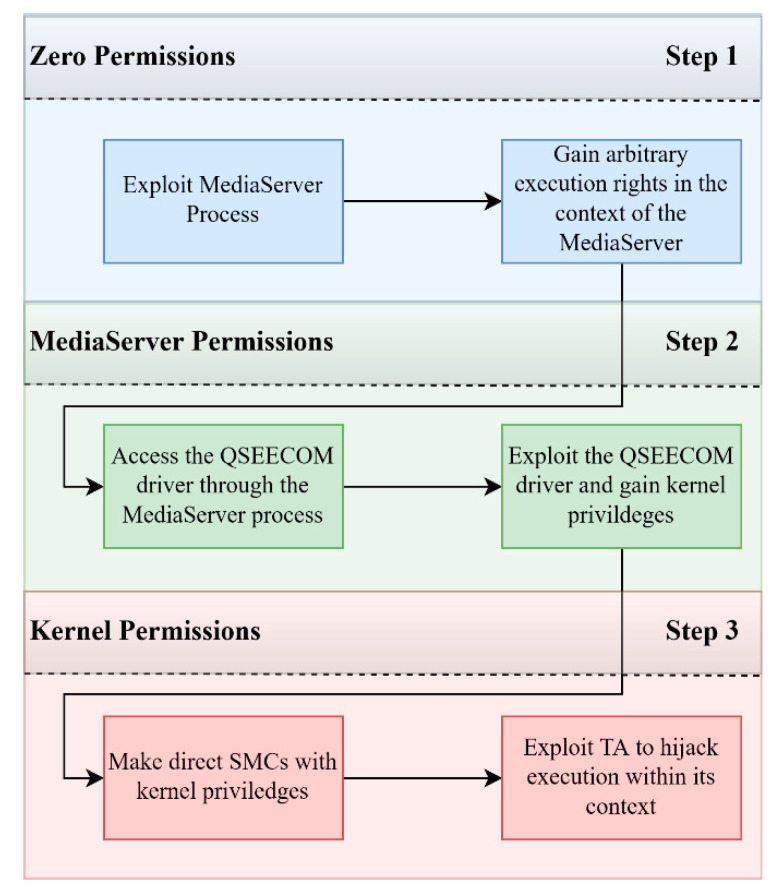
Privilege escalation to TrustZone.

First, we describe a set of attacks that target the QSEE and come from the same researcher [82,83,84]. The feasibility of these attacks has been demonstrated through the development of exploitation code. The first attack was published on [82] and is composed of three basic steps as shown in Figure 6. In the first step, the attacker, with zero permissions, exploits a vulnerable implementation of the MediaServer Android application running in the normal world. As mentioned previously, MediaServer has the privilege of accessing the qseecom driver in order to communicate with the TEE and in turn with the WineDive trusted application. In the second step the attacker, through MediaServer, gains control of the Linux kernel by exploiting a vulnerability in the qseecom driver (which runs in the context of the normal world kernel). At this stage, he could perform direct SMC calls to the secure world through the privileged standpoint of the normal world kernel. Finally, in the third step, the attacker achieved arbitrary code execution in the context of a trusted application that he exploited by having direct access to it through SMC calls. While the first two steps and their vulnerabilities were not related to TEE (and hence we do not analyze them), the third step exploits a vulnerability in the QSEE.

**Observation 1:** The TEE can be directly accessed from kernel privileged applications through SMC system calls. This provides a significant attack vector since privilege escalation attacks are abundant allowing for potential exploitation of the trusted world.

More specifically, in the third step, first the attacker discovers that one of the several available SMC calls, does not perform memory checking allowing to write a zero DWORD in any specific memory address (zero write primitive). This vulnerability stemmed to the fact that the attacker was able to call a trusted application that uses a shared memory through an *scm_call_atomic()* function. More specifically, the trusted application expected to write the (zero DWORD) result of its operation to a specific memory location which is now controllable by the attacker as it is a parameter in the *scm_call_atomic()* function. This zero write primitive is used to disable the memory address checking mechanism that is used to validate each memory address that is passed to the secure world.

By disabling this operation, the attacker is able to exploit other SMC calls in order to create different kinds of primitives. In particular, after the invalidation of the control mechanisms, the attacker now is able to utilize SMC calls to upgrade the zero-write primitive to arbitrary write and read primitive. Now that the attacker has arbitrary write capabilities, the problem is to identify executable memory regions to write his own shellcode in. TrustZone kernel code pages are mapped as read-only, thus no modification is possible without first circumventing this protection. A specific register within the ARM MMU, the Domain Access Control Register (DACR) is responsible for the protection of TZ memory, by controlling which memory regions are accessible for reading and/or writing. By utilizing the aforementioned arbitrary write primitives, a modification to the value of the DACR is made (the entire DACR is set to 1, see Section 2.1) that enables read and write capabilities to all controlled memory regions effectively nullifying the mechanism. With this done, the attacker can now write on any memory region that is not protected with the XPU mechanism. To finally achieve execution, he wrote his shellcode in some identified “code caves” within the kernel which are executable allocated memory regions that are unused by the kernel and altering them will not affect it.

##### TrustZone Kernel Exploitation

**Figure 7:**
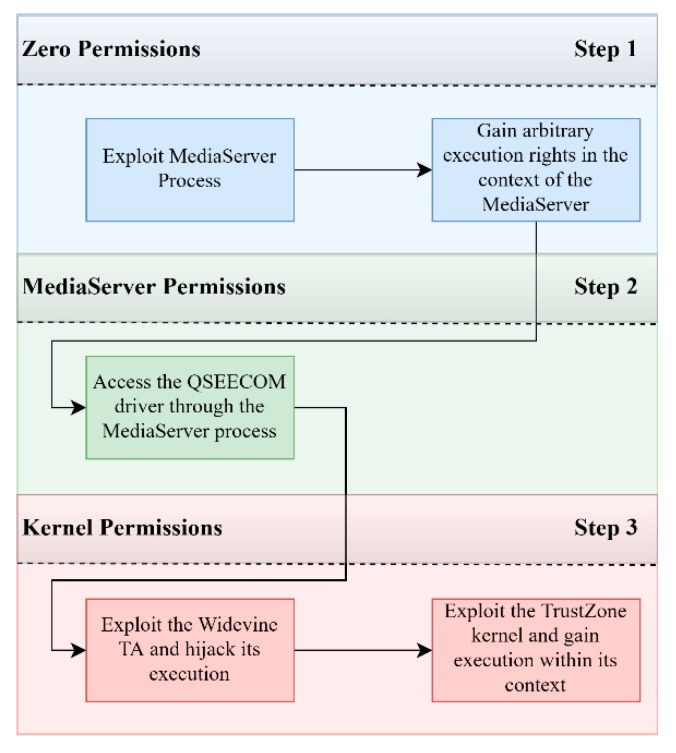
Privilege escalation to TrustZone kernel.

The second published attack [71,83] by the same researcher, provided an exploitation chain that could let the attacker reach TrustZone kernel privileges. The researcher followed a different path and chain of exploits than the previous attack, as he skipped the Linux kernel exploitation altogether and escalated his privileges in the TrustZone from user to kernel. In particular, the attack is composed of three stages, ranked by the level of privileges the attacker achieved (see Figure 7. The first two stages are identical to the previous attack with the only difference being that instead of exploiting the user-space kernel through the MediaServer application, he directly used the qseecom driver since this application is privileged to access it (see Section 2.4).

**Observation 2:** The TEE can be indirectly accessed through the privileged applications that can communicate with the TEE driver and in turn with the TEE itself. These applications must be bug-free and well protected.

The MediaServer application can only communicate with the Widevine trusted application which is responsible for DRM media playback. The exploitation of the Widevine trusted application was based on a memory copy buffer overflow in an unused function named *PRDiagVerifyProvisioning().* Due to the buffer overflow, an arbitrary write primitive is achieved, which allows the execution of arbitrary code within the context of the trusted application.

**Observation 3:** The TEE does not implement classic security measures such as ASLR. This allows for common code execution and privilege escalation attacks that could have been avoided.

**Figure 8:**
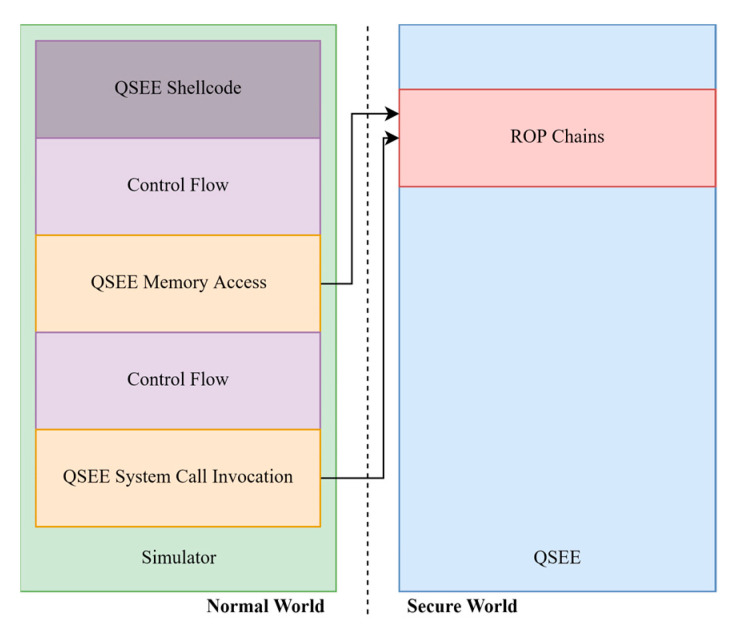
Emulating QSEE code and partially offloading it to QSEE.

Since the executable code segments of the trusted application are mounted as read only, the attacker had to resort to a ROP chain to run his code, something that turned out to be infeasible. To bypass this problem, he segmented the code execution in two parts, the normal world part, and the secure world part. Any piece of code logic that did not need QSEE privileges was emulated within the normal world, and only when the intended functionality needed to be run within the QSEE, then small ROP chains were invoked to execute these special cases, this is depicted in Figure 8. The only functionalities that needed these elevated privileges were only two:

(1) Reading and writing memory (QSEE Memory Access)
(2) Calling system calls exposed by the TrustZone kernel (QSEE System Call Invocation)

Practically, what the researcher did here, is that instead of creating large and possibly infeasible ROP gadgets, he divided his exploitation code into a simple execution segment which included common ARM opcodes and a QSEE segment which included opcodes that could only run within the QSEE environment. This way he only needed to write ROP gadget chains for the QSEE functionality of his exploit, the rest was run in the normal world where he could execute any command without resorting to ROP. At this stage, he was limited to code execution in QSEE user-space but had no TrustZone kernel privileges. The only attack surface to the kernel appeared to be through the system call functions exposed by the TrustZone kernel to the trusted applications. For this reason, in the third step the attacker exploits the WideVine Trusted application to pivot and exploit the TrustZone kernel.

Similar to the normal world applications, trusted applications running in secure world user-space can invoke system calls exposed by the TrustZone kernel, by issuing a specific instruction named SVC. In the normal world when a system call is performed the code execution is transferred to the Linux kernel. Similarly, when a trusted application performs a system call the execution flow is transferred from the user space trusted application to the TrustZone kernel to handle the SVC request. In particular, when an SVC instruction is called the TrustZone kernel uses the VBAR register to handle the SVC requests and find the address of the appropriate kernel exception vector. The system call handling in the TrustZone kernel accepts as input the arguments of the system call as it was issued by the user space trusted application running in QSEE. However, a critical observation here was that the TrustZone kernel does not perform any kind of checks on the validity of the provided input buffers, meaning that it accepts all provided arguments in system calls coming from legitimate trusted applications as valid, despite the fact that there exist some basic sanity checks of the input.

With this in hand, the researcher identified an exploit that allowed him to force the kernel to use an attacker-made exception vector table (instead of the legitimate one) and thus the attacker was able to overwrite any exception address with a pointer to any other function (system call hijacking). The only objective remaining, is to find a writable and executable memory region to write the shellcode that the hijacked function pointer will point to. The trusted application code segments are perfect candidates, besides the fact that they are write protected by the DACR mechanism since it can conveniently be disabled with the system call bug from the Privilege Escalation to TrustZone attack described previously. With the DACR memory protection disabled, the researcher was able to write any shellcode in any code segment of the trusted application and invoke it in the context of the TrustZone kernel by calling the modified system call handling function and thus achieving arbitrary code execution with secure world kernel privileges.

**Observation 4:** The TEE kernel in many cases blindly trusts the trusted applications since they are considered to be untampered and secure. This could provide an attack vector if a trusted application is successfully exploited.

##### Extraction of Master Keys

The final attack that was described by the same researcher [84] extends the previous ones and demonstrates the ultimate impact that an attack to the secure world can potentially have in an Android system. This attack is a chain of exploits utilizing the capabilities that were gained from the previous two attacks described in Section 3.1.1 and Section 3.1.2. It allows the extraction of any TEE protected key, such as the Full Disk Encryption (FDE) key of Android, thus allowing the decryption of any device encrypted disk that contains a vulnerable version of the WideVine trusted application.

By reverse engineering the KeyMaster trusted application, the researcher discovered that the FDE key (i.e., a symmetric key used for disk encryption) is not protected by a hardware-bound key but instead from a key which is derived from a hardware-bound key and stored in the global buffer of the KeyMaster trusted application. This means, that it is protected from other trusted applications (trusted application isolation), but it is accessible from the TrustZone kernel. Based on this observation, the researcher was able to execute code inside TrustZone kernel (with the primitives established from the previous sections) that would extract the FDE key from the KeyMaster trusted application, breaking this way the security of the full disk encryption scheme in Android.

**Observation 5:** Even with the security guarantees of TrustZone, critical components such as the FDE key can be extracted if the kernel of the secure world is compromised.

**Figure 9:**
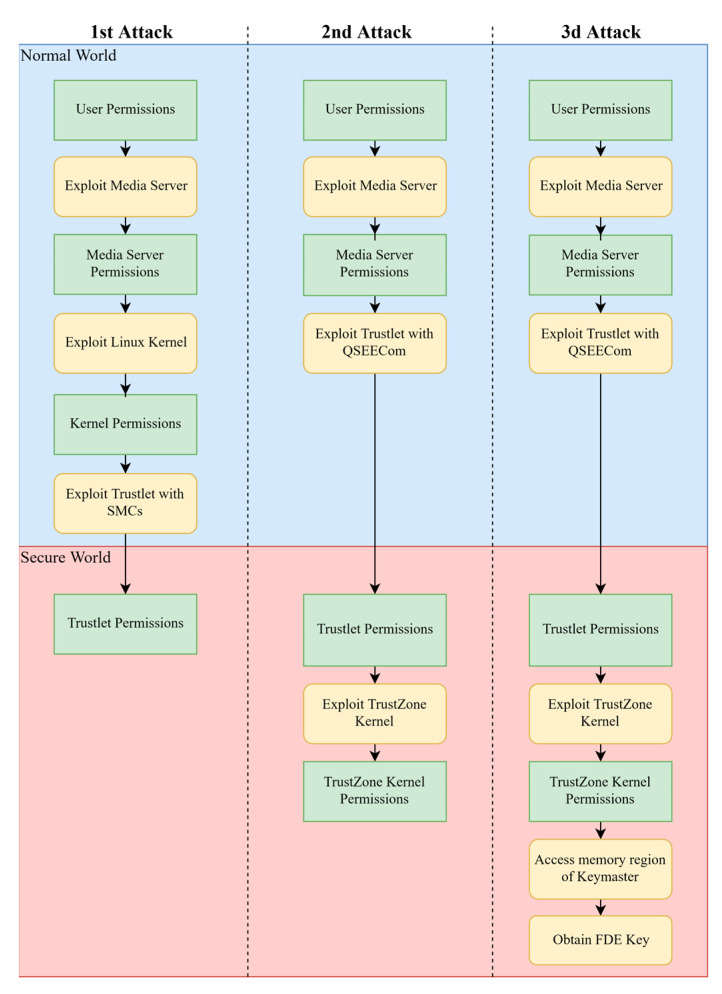
Three steps to complete TrustZone exploitation of the bits please attack.

The researcher has noted that this attack was possible because the encryption of the disk was not based on a hardware-bound key but on a software key. This, in combination with the already achieved TrustZone kernel exploit, enabled this attack which ultimately broke the FDE scheme of Android. A key remark that the researcher makes, is that the full disk encryption system of the android is only as strong as the security of the TrustZone kernel or the KeyMaster trusted application, as a problem with any of them will potentially leak the master key of the FDE. Finally, an overview of all the aforementioned attacks is depicted in Figure 9. As it can be seen each attack is building upon the previous attacks in order to achieve higher privileges within QSEE.

#### 3.1.2. Unbox Your Phone (Buffer Overflow and Overread-Based/Logic-Based)

**Figure 10:**
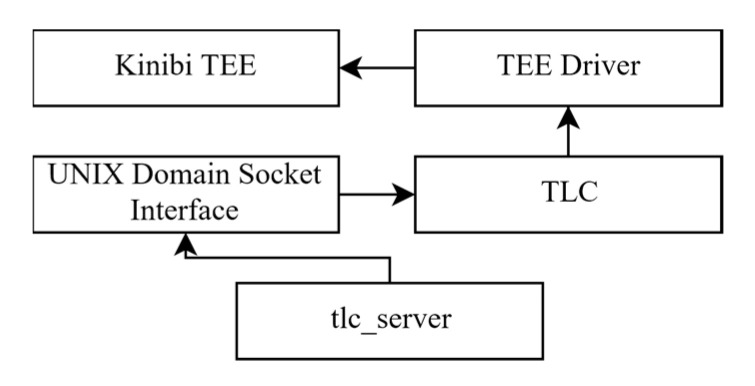
Kinibi TEE communication scheme.

Until now we have seen a set of attacks on a TrustZone-based Qualcomm implementation of a TEE. A security researcher [11,85,86] discovered a set of major vulnerabilities that affect another widely adopted TEE, the Kinibi TEE developed by Trustonic, which is also based on the TrustZone technology. Like the previous attacks, the problem is rooted in the code implementation of a trusted application, but it was made available to the attacker due to inadequate protection of the communication between the normal world and the trusted world. More specifically, Trustonic has implemented an overlay named TLC (trustlet connector) that acts as a gatekeeper to Kinibi. The TLC exposes an interface to the normal world userland, that in turn will expose an interface to the normal world to use via UNIX domain sockets (see Figure 10). These domain sockets make sanity checks on TEE requests, enforce access control through MAC/DACs schemes and are further protected through selinux with the only entities having access to them being some Samsung implemented proxy applications. The proxy application that the attacker targeted is called “tlc_server” and it handles the communication for five supported trusted applications.

First, the researcher identified an authentication bypass in the tlc_server binary by finding out that all commands exposed by this application implemented a way to verify the caller’s permissions except for one command that did not include such a security mechanism (Vuln 0). This way any user-space application could use this handle to open a session to a trusted application and send arbitrary commands to it. Through this authentication bypass, the attacker gained access to the ESECOMM trusted application that implements a secure interface to an embedded secure element with the use of the “SCP03 Global Platform Secure Channel Protocol”. The messages that are sent through this protocol are APDUs (application protocol data units) which are encoded in TLVs (type-length-value). There is a utility function in this trusted application that parses these TLVs that although makes some sanity checks on the TLVs themselves, it fails to check if the number of TLVs exceed the number of max TLVs that can be stored on each structure. By sending a number of TLVs that exceeded the capacity of the structure, the researcher was able to orchestrate an overflow attack (Vuln 1). Moreover, these structures are allocated on both the stack and the heap, providing a variety of available attack paths. Additionally, the TLV parser fails to properly bound check the reading from the input buffer that contains the TLVs (it checks if the offset is equal to the end of the buffer instead of if it is lower than it) and it can be trivially be bypassed to read beyond it (Vuln 2).

Another stack buffer overflow is present within the ESECOMM trusted application in a function called “parse_ca_cert()”. This function contains a textbook buffer overflow, as it fails to check the length of an input value from a TLV and it copies it to a 32-byte buffer. Although there is a restriction that TLVs should not exceed 0 × 400 bytes in size, it is not enough to mitigate this attack as the buffer is only 32 bytes in size (Vuln 3). There is a similar vulnerability in another function named “parse_scp_param” which is used to parse the secure channel parameters in order to establish a secure channel between Kinibi and the secure element. This function parses and copies several parameters for the Diffie-Hellman key exchange functionality. All of these parameters are sanity checked except for one parameter that is partially checked that allowed the attacker to establish another overflow attack (Vuln 4).

In contrast with the previous attacks, the next attack requires that the user has root privileges in the normal world, but it provides by far the largest attack surface as it can be exploited from many places within the code. In order to exploit this bug, the attacker must bypass the tlc_server since it includes input sanitization that prevents this attack and should use the block device driver (/dev/mobicore) directly by exploiting one of the many applications that have access to it. The problematic behavior lies on the parameter that is used to specify the memory offsets of the request and response messages within a common buffer that the two worlds share (named TCI). This TCI buffer contains a field named “envelope_len” that defines the offset within the buffer that the response message should start. This field is set by the tlc_server or any other valid applications and it is inherently trusted by most trusted applications, if an attacker obtained root privileges, he could set this field to any value he wanted and thus force a trusted application to write or read on any given memory (Vuln 5).

The researcher exploited this bug by setting arbitrary envelope_len values in order to specify the offset that he wants to write to, even if this offset is beyond the limits of this buffer. This way a semi-controlled (only the address offset is controlled, not the data to be written) write primitive to the secure world is found, that could be fully controlled if a trusted application that has user-controlled output is found amongst the trusted applications residing in the TEE.

As it can be seen in Table 1, the researcher managed to find six vulnerabilities in total that where all documented and submitted as SVEs. This number of vulnerabilities should be indicative of the attack paths that an adversary might be able to take when he targets a TEE. All these vulnerabilities where available due to software bugs that could have been avoided with proper coding techniques.

**Observation 6:** Code bugs are abundant in applications. Special care must be taken when developing applications that belong either to the trusted or world or to the normal world.

**Observation 7:** Since code bugs cannot be completely avoided, critical applications must not contain a single point of failure. On each application layer there should be security and sanity checks that will cripple an attacker if he manages to bypass any previous layers.

#### 3.1.3. Other Software Attacks

Software attacks are common against TEE implementations, for example, the attack documented in [87] (Logic-based) demonstrates how a failed memory validation leads to an arbitrary read and write exploit in the secure world. Similarly, in [13] (Logic-based) a flawed SMC memory checking mechanism allowed a kernel-privileged attacker to write controlled data to arbitrary secure world memory. On another occasion [88] (Buffer Overflow and Overread-based) an overflow attack allowed the researcher to achieve arbitrary code execution within the secure word. Besides software bugs, there are other attack vectors such as bad usage of cryptographic algorithms, in [89] (Bad Use of Crypto) the researcher discovered that the secure boot process used in a TEE protected device was only based on SHA256 to verify the integrity of the system which allowed him to modify and load any piece of software completely bypassing the secure boot.

In [90] (Logic-based) another vulnerable SMC function exposed by QSEE was discovered. Because of a vulnerability in the handling of input of SMC calls again - in the specific case a signed comparison on unsigned user input bug ‒ an attacker could write zeros (0) to a secure memory region of his choice, bypassing that way security mechanisms and finally gaining arbitrary read/write capabilities in the context of the TrustZone kernel. This vulnerability affected at current time all devices that used the Snapdragon 805 SoC and utilized QSEE for TEE. On another occasion, Di Shen [29] (Logic-based) published an attack affecting HiSilicon’s TEE, another implementation less widespread than QSEE found in found in Huawei devices. The researcher discovered a vulnerability that allowed him to issue arbitrary SMC calls which led him to a secure world arbitrary write gadget. By subverting security protections that were in place he managed to achieve code execution in the context of the secure world. This attack enabled him to gather sensitive fingerprint data from the secure memory region.

On another occasion [91] (Buffer Overflow and Overread-based), the researcher managed to find serious security bugs within the ARM trusted firmware image installed in a commodity Android smartphone. This has allowed him to run code in ARM Exception Level 3 (supervisor) which has higher privileges than the TrustZone TEE. With this in hand he was able to completely bypass the face ID authentication mechanism that runs within the TEE by patching the binary of the trusted application so that it accepts any face as a valid authenticated user.

In [92] (Buffer Overflow and Overread-based/Logic-based), several security flaws were identified in the Huawei TrustedCore which used easily bypassed white-box AES for the encryption of the trusted applications which allowed the researchers to decrypt them and reverse engineer them. Through further analysis, they discovered a design flaw in the KeyMaster trusted application that used a constant key to encrypt the master key and extract it. Finally, they discovered a buffer overflow in the KeyMaster trusted application, which allowed them to execute code within the context of the TEE kernel.

### 3.2. Architectural Attacks

#### 3.2.1. Boomerang Vulnerability (Unused Security Features)

This class of vulnerabilities [14] arises due to a fundamental flaw in the design of the communication between the rich and the trusted operating system (OS). The impact of BOOMERANG is that an attacker can gain arbitrary read and write capabilities, on any memory location in the rich OS, by using the high privileged trusted OS as a proxy. With these gadgets available, the attacker can bypass the sand-boxed memory of the Android system and retrieve data from other applications, obtain root privileges, and potentially gain full control of the rich OS. The research has shown that this problem extends through hundreds of millions of devices that are currently on the market.

The exploitation is possible due to the fact that the trusted OS can read and write any memory address of the rich OS, without taking into consideration its access rights properties due to its high privileges. In more detail, both operating systems have mechanisms in place to sanitize memory addresses and protect them from unauthorized access from within the OS, the rich OS protects its memory from normal world applications and the trusted OS from trusted (and normal) applications. The problem arises when the system performs cross OS memory access, the untrusted environment has no authorization to act upon trusted memory, as expected. The trusted environment, on the other hand, can access any memory address given to it and has no way of determining the security properties that exist in the normal OS or the provenance of this address. As a result, the trusted OS, since it does not check the privileges of the rich OS caller, will perform any action on any memory address blindly. The method used to avoid the security measures in place is implementation dependent, the researchers have successfully attacked a variety of device architectures that cover a large proportion of the available devices on the market.

The actual attack is performed from the non-secure world, where an application or user passes the malicious address as a variable to a secure world call. Due to the lack of message exchange standardization, not all addresses passed to the trusted application are monitored and as a result, not sanitized. This way an attacker can read or write on any memory inside the non-secure world and depending on the available trusted applications, he can create gadgets that will write specific data which can potentially lead to full exposure of the normal world. The attack, according to the paper, was successful against the QSEE trusted execution environment by using widely deployed trusted applications, such as the KeyMaster, WideVine, and PlayReady. When vendor-specific trusted applications are taken into consideration the attacker is provided with many more trusted applications to choose from. The researchers, using a Huawei device, have gained root access on the device by using arbitrary read and write functionality gained this way.

**Figure 11:**
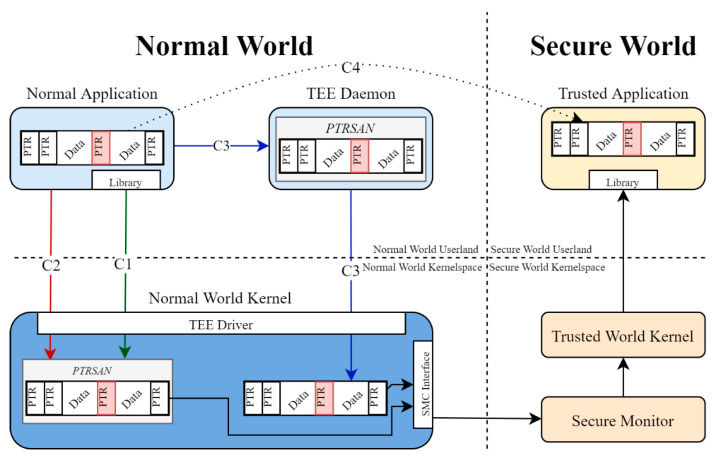
Boomerang attack architecture.

Figure 11 explains the attack in a high level. The attacker wants to transfer the malicious pointer to the trusted application (C4). In order to do that, he populates a structure with the pointer without annotating it to avoid the sanitation process. The data is then passed to the underlying supervisor mode by using three possible routes: a TEE Daemon (C3) that runs on the background and also handles pointer sanitation, an API that can be used directly from within the application (C2) and the most implemented way that uses a library to provide the aforementioned API (C1). In the cases of C1 and C2, the pointer sanitation is done in the normal world kernel instead of the TEE daemon. The normal world kernel will now make an SMC call to change worlds and transfer the data structure to the secure monitor which in turn will pass them to the trusted world kernel. The data structure is now in the trusted kernel which will check if the pointers within the structure refer to memory inside the secure world. Since the malicious pointer’s provenance is the non-secure world, it passes the test besides the fact that it can potentially point to normal-world kernel memory. Finally, the trusted kernel passes the structure to the trusted application which will act on the given non-secure world address without any further checks. It is obvious that by the time the attacker passes the normal world pointer sanitation, there are no other security checks in the way and since the secure world has absolute privileges on normal world memory, it can access and manipulate any given pointer.

**Observation 8:** The TEE can be utilized as a primitive for attacking the untrusted world due to its high privileges. Even without exploiting the secure world, it can be misguided into modifying memory zones in the normal world which otherwise would be inaccessible due to the high privileges of the TEE.

The privileged standpoint of TrustZone TEEs has been identified from 2013, in [93], where the researcher proposed a powerful rootkit installed in the secure word. The secure world has total access to all memory, allowing modification of the normal world kernel structures providing the basis for a rootkit that has access to every part of the system. Additionally, TrustZone is able to isolate its state and block access from the normal world to its own memory, effectively concealing the existence of the rootkit. Although the researcher does not present a specific implementation, he proposes the use of various techniques to cloak the visibility of the code running in the secure world and make its detection even harder. Essentially the secure world provides all the requirement for an attacker to implement a malware that can control the whole system and be unable to be detected in doing so. Furthermore, in [94] the researchers also investigated the possibility of concealing and checking the integrity of a hardware-assisted rootkit.

#### 3.2.2. Downgrade Attack (Unused Security Features)

This kind of attack was found to be effective against many ARM TrustZone-based TEEs that are widely deployed [95]. The root of the problematic behavior is in the trusted application verification by the secure world and on how the TEE checks for the version of the trusted application. This research has presented that an attacker could potentially load older versions of a trusted application to the system and execute them instead of the already installed ones. This way, a system is vulnerable to any past found and fixed problems that might have occurred since the attacker can just load the vulnerable trusted application and exploit it.

When a vendor is aware of a flaw in one of his trusted applications, he can issue an update that will fix the problematic behavior. The new application is accepted into the system since it is signed with a private key and verified with a corresponding public key trusted by the TEE. The presumed behavior is that the system, after the update, will not accept an old version of the trusted application, contrary to that, the system will accept any version of the trusted application as legitimate and let it run, since it is also signed with a correct private key. It is noteworthy that most of the TEEs have mechanisms to version control their trusted applications (and thus fix this vulnerability), but according to a blog post by Gal Beniamini [9] all the trusted application binary images (45 different firmware images) that he analyzed had a version number of zero, even the updated ones. It is obvious that if the version number is not incremented, the TEE will accept any version of the trusted application believing it is in the same version.

With that in mind, it is obvious that an attacker that has access to older and vulnerable versions of trusted applications, in conjunction with the necessary root privileges (not always required), can overwrite the up to date and secure trusted applications and exploit flaws that existed before the fixes. With this attack available, it is mandatory that trusted application developers should employ the version control mechanisms that the TEE manufacturers provide.

**Observation 9:** Developers are not fully utilizing the security features of the TEE. Even if most TEE implementations provide version-based blocking for their trusted applications, it was found that the developers did not use this functionality. This has led to a major architectural vulnerability that allowed attackers to load old versions of trusted applications.

#### 3.2.3. Vulnerabilities in a Heterogeneous SoC (TrustZone Implementation-Based)

From 2000 until today companies have anticipated the need for more advanced FPGA functionality that could come from integrating SoCs in their model. A heterogeneous SoC is a system that combines a FPGA, a SoC and other intellectual properties (IP) and devices. Having the security of TrustZone in hand was very important for FPGAs since they are actively used to handle sensitive data in cloud infrastructures. For example, FPGA-based SoCs are used for secure data processing, to implement internet protocol security (IPSec), to secure communication in software radio systems, to secure cyber-physical infrastructures, industrial control systems and safety-critical systems [96]. The question raised by the paper [97] is whether the security provided by the TrustZone is propagated through the variety of IPs in the system and illustrated by a number of theoretical examples. The communication between the various devices and the IPs within the programmable logic partition of the FPGA is established through a programmable interconnect called Advanced eXtensible Interface (AXI) that multiplexes the communication between the IPs and the devices. The IPs are divided into secure and non-secure and depending on the access rights of the requestor, the AXI allows or prevents him from accessing the IP. All the attacks are based on the ability of the attacker to modify the design of the FPGA and the AXI, there are six attacks demonstrated:

- The first attack changes the design in order to modify the security bits of the request and make the AXI think that the requestor is a secure application and as such has access to all IPs regardless of their security level.
- The second attack is a DoS attack that is the reverse of the previous attack, it sets the security bit of every request to non-secure so that any legal request from the secure side to a secure IP is denied from the AXI.
- Like the previous attack, the third attack forces to programmable logic (PL) part to always have a positive response to IP requests. Any non-secure request to a secure IP will have a false positive response when the process should have stopped with an error.
- In the fourth attack the PL is configured to return an error on every access, so that all the IPs connected to the specific AXI will be unusable.
- The fifth attack is different from the previous, as it does not incorporate message manipulation. A FIFO buffer is injected inside the AXI interconnect within the PL and configured to spy on any transaction the AXI handles, especially secure transactions between secure components.
- In the final attack, a malicious IP with a memory mapped master port is designed into the FPGA. With the master port, the IP will have direct access to secure memory with sensitive data. In order for the attack to work, the mapping of the master port should cover the memory containing the information to be extracted.

In [96], the researchers have extended the research around these attacks with a more detailed analysis of these security issues. Furthermore, they propose possible mitigations that could be applied across the system (SoC, FPGA and AXI) while they underlined the need for the integration of security in the FPGA design process.

#### 3.2.4. Other Architectural Attacks (TrustZone Implementation-Based)

On another occasion [98,99], the debugging feature of ARM named CoreSight was the enabler of a series of attack not only to the SoC in general but also in the TrustZone TEE installed in the system. More specifically, the researchers managed to extract fine grained information from the trusted world that allowed them to completely reverse the AES encryption process that was executed in the TEE. This way, the key of each AES round was discovered which ultimately exposed the core encryption key that was used in the process. Additionally, the researchers were also able to reconstruct images of the fingerprints stored in the TEE, exposing very sensitive and non-revocable authentication data.

Additionally, a fundamental of architectural attack [100,101,102] was made publicly available against the gaming console Nintendo Switch. The main issue of this vulnerability is a core architectural design that affected the deep sleep state of the console which failed to validate the saved state of TrustZone in the device memory. This way the attackers were able to change the saved state while also changing the MAC and the key used in the MAC process. As a result, the device would wake up with a changed but valid TrustZone state leading to a complete compromise of the trusted world.

### 3.3. Hardware Attacks

#### 3.3.1. Side Channel Attacks

Side channel attacks [75], are a family of attacks that gather leaked hardware information in order to gain sensitive data from a system. In essence, as their name indicates, they utilize a side channel to obtain information instead of the standard channel that might contain security roadblocks. When a process is running a cryptographic operation, an attacker can measure and accumulate information such as: cache access attempts, timing computations, power consumption, electromagnetic leakage and even the sound produced during a computation. It is through that hardware information that an attacker can gain sensitive data and expose cryptographic keys.

The cache timing attack is a side channel attack and consists of two phases, the timing phase where the attacker sends plain text, known or unknown, to a cryptographic implementation and then measures the time it takes for each encryption. In the second phase, after enough plain texts have been encrypted, the attacker correlates the measurements and ends up with a much shorter key space that can be brute forced easily or even the key itself.

The attack is based on a core CPU architectural design, the use of the cache. More specifically, the CPU uses the cache to store data for quick access, if the data it needs are contained within the cache, then we have a cache hit, if not a cache miss. Depending on how many cache hits or misses happen during the execution of a sensitive operation, the total run time could be affected greatly. After enough samples are collected, an attacker can correlate the inputs with the time taken for each execution and deduce the key. This method is the simplest, there are more advanced methods like, evict + time and prime + probe, that actively manipulate the cache in order to produce data with more entropy that can provide results with a smaller data set.

There are many cases where these attacks were successful against Intel x86 CPUs but three cases [12,73,75], have shown that ARM-based CPUs are affected just as much. Weiß et al. [75] implemented an attack on a virtualized ARM system that contained two isolated worlds, although it was not a TEE, they have proven that cross-isolation attacks are possible. On the other hand, Lipp et al. [12] have successfully attacked consumer Android devices with advanced cache timing attacks and have shown that TrustZone technology does not protect the system from cache timing attacks. Finally, Zhang et al. [73] were also successful in applying their attack named “TruSpy” against a TrustZone implementation and have proven that it is possible for an attacker coming from the real world to steal secret information from the secure world isolation using a timing-based cache side-channel. We will analyze the core concepts of side channel attacks found in the literature.

##### Cache Timing Attack

The attack presented by Weiss et al. [75] is the first indication that cache-based timing attacks could be utilized to break virtualization barriers. That is why the researchers have emulated a TEE by creating a virtualization environment where two worlds exist just like we have seen in the TEE so far. They have installed an AES based mutual authentication protocol that is resistant to replay attacks with all the encryption being done on the secure side. This authentication scheme is the target of the side channel attack that will ultimately reduce the key space so much that brute forcing the key will be trivial.

**Figure 12:**
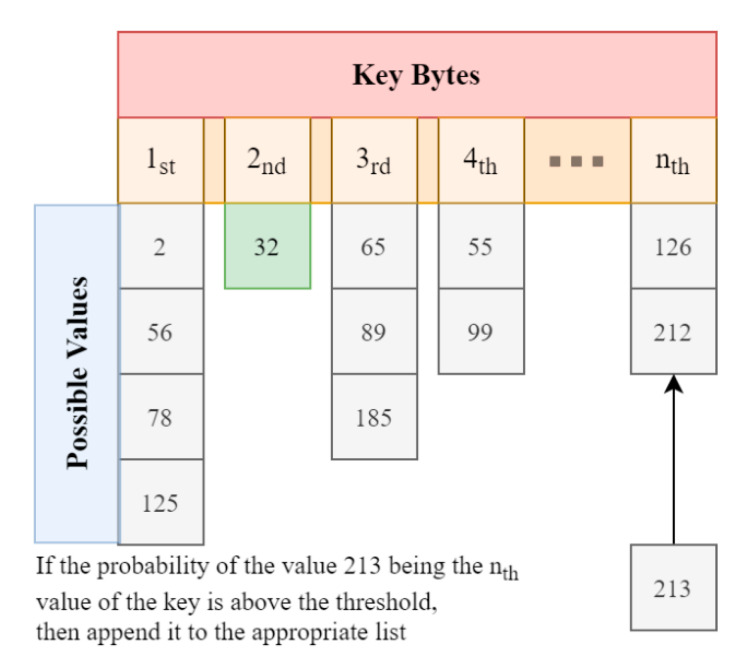
Generating the list of possible values for each byte of the key.

The attack that the researchers have decided to implement is a basic timing attack as it is the most general possible attack that could be applied to almost all devices. They have separated the attack in two stages, the offline and the online phase. They first begin with the offline phase, where they proceed to collect timings of multiple encryptions using a known, all zero key. This phase will produce data that will later be correlated with the data collected from the online data set. In the next phase, the same procedure takes place but this time the key is unknown. After enough timing data is collected, the two compiled data sets are correlated with the final product being the possible values for each byte of the key. These values are generated based on a probability threshold, that is, if a byte of the key has a chance higher than the threshold to hold a specific value, then this value is added to the list of possible key values, if not then it is left outside of the list (see Figure 12).

The attack was implemented on an ARM-based development board named Beagleboard with an L4 microkernel as a virtualization layer. The board has an ARM Cortex-A8 which has been used widely in smartphones when the research paper was being developed. The researchers have timed the execution time of each encryption precisely with the usage the ARM CCNT register, which holds the total clock cycles of the CPU since the last reset. With all these in hand, they applied the attack to several AES implementations with most of them being vulnerable to some degree.

##### Prime + Count

**Figure 13:**
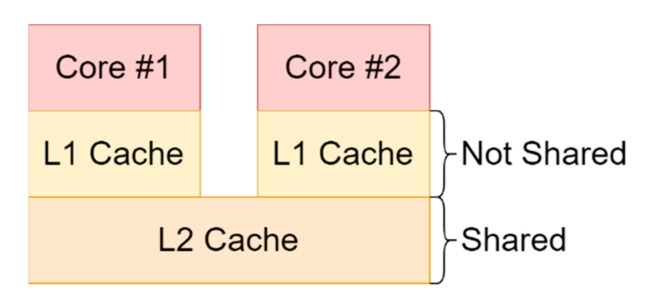
L1 & L2 cache architecture.

The prime + count attack is quite different from the previous attacks, as it is not as fine grained, but it promises reduced noise levels introduced by the TrustZone world switching mechanism and pseudo-random cache replacement policies. The proof of concept presented in the research [103] is a data exfiltration scenario and it is composed of a sender that encodes a message and writes it accordingly to a shared cache and a receiver that primes the cache beforehand and reads the message that was written from the sender. The attack has two versions, the single-core, and the multi-core, with each of them being applied to a different cache level. There is a L1 cache for each core of the CPU and it is not shared by other cores, while the L2 cache is much larger and it is shared amongst all cores (see Figure 13).

We will first focus on the steps of the single-core scenario first. In the first step of the attack, the receiver primes the cache by invalidating all its entries and loading specific data to fill the entire L1 cache. After that, the control is given to the sender application in the trusted world where it encodes the message to be sent and proceeds to invalidate entries and write data to the L1 cache according to the encoded message. Then the normal world takes back the control and counts the number of the cache accesses that happened during the trusted world execution time and through that information it infers the message that the sender application sent. The multi-core attack is quite similar as the only difference is that the prime phase fills all the L1 and L2 caches while the sender writes its messages on the L2 cache. It is obvious that the second attack is much more difficult to apply, as the L2 cache is a global cache and as such it will introduce noise from parallel applications that execute in other cores.

The messages are encoded according to the number of cache accesses, for example if the sender wanted to send the number 20 it might have been agreed that 40 cache accesses should be made. To clear any noise on the channel, a method that the researchers named “bucket” was used that practically is a more flexible decoder. If the decoder received a number of cache accesses in the range of 40–50 then it would understand that the sender intended to make just 40 accesses and translate that to the number 20.

The prime + count attack cannot be used as an as is attack to spy on the TrustZone, as it is not able to access sensitive information and deduce the exact values that were written to the cache. This attack is basically a proof of concept that there could be a side channel established between the normal and the secure world just by encoding messages using the number of cache accesses as a primitive. According to the research paper, the bandwidth could be as high as 27 KB/s in the single-core scenario and 95 B/s in the cross-core scenario.

##### ARMageddon

In this work [12] there is a demonstration of how side channel attacks could be implemented using only unprivileged applications. The researchers have presented effective ways to apply many cache-based attacks like Flush + Reload (which is used in a similar manner in [104]), Flush + Flush and Evict + Reload on commodity ARM based Android devices. The main challenges that were tackled are the following:

The fact that the last level caches (L2 for ARM) of ARM-based CPUs are not inclusive. That means that entries on lower levels of the cache are not guaranteed to also exist on the last level caches that is shared amongst all the cores of the CPU. This fact introduces a problem when cross-core attacks are to be made, as the last level shared cache is the only means the attacker has available to access and manipulate data from other cores of the CPU. The paper presents a novel way that exploits cache coherency protocols and transfers between L1 and L2 in order to “patch” the last level cache non inclusiveness problem.

Furthermore, modern devices employ multiple CPUs on their design that do not share a common cache between them. However, the cache coherency protocols are used to fetch lines cache entries between different CPUs quicker than from the main memory. The researchers exploited this design to mount attacks that are effective even in cross-CPU environments.

Another problem was that most of the ARM CPUs do not support the flush command on which the Flush + Reload and Flush + Flush attacks are based on. The researchers have investigated over 4200 cache eviction strategies based on Rowhammer techniques in order to find the best alternative for the missing flush command.

Additionally, ARM CPUs employ a pseudo-random cache replacement policy that makes predicting which cache line will be replaced difficult. This fact produces needless noise in the measurements of the cache during an attack. The researchers proposed a methodology that reduces the effects of erroneous prediction of replaced lines in the overall performance of the attacks they implemented.

Accurate timings of execution require access to functions that are only available to the kernel, since the attack model that the researchers chose does not include a rooted device, they had to find alternatives that provided timings that were capable to allow the attacks to happen. They proposed three different solutions: a performance monitoring system call which was available to the user-space, the POSIX function that returns the time and a dedicated function developed by the attacker. All of those methods where successful in identifying cache hits and misses and thus are viable for side channel attack implementations.

The presented attacks were made against three off the shelf devices with each one having different CPU ARM-based architectures. They first created a sender and receiver that utilized the attacks in order to pass messages and measure the throughput of their implementation in a data exfiltration scenario. Their attacks had a minimum transmission rate of 12.5 kbps and a maximum of 1140 kbps depending on the attack implementation and the underlying hardware. Furthermore, they implemented actual attacks to spy on other processes that use shared libraries of the system or android applications that run on the android runtime ART. They have also shown classic attacks on cryptographic primitives such as AES with a t-table implementation. Finally, they show that their attacks can also be applied over the boundaries of TrustZone although they implemented very simple proof of concept attacks against it. This shows once again that the TrustZone technology is susceptible to cache-based side channel attacks since the cache is shared amongst the secure and the normal world.

**Observation 10:** The TrustZone TEE since it is implemented on top of the main CPU and it does not utilize separate hardware, shares the cache of the CPU with the normal world. This has created the possibility of side channel attacks which could produce devastating attack vectors such as private key leakage.

##### TruSpy

The TruSpy attack [73,105] was the first that provided an actual proof of concept for a cross world side channel attack. The researchers have presented two separate attacks that both achieved to break the word separation barrier with each attack having different privilege requirements in the normal world (i.e., kernel and user privileges). The kernel privileged attack is easy to implement since the attacker will have access to the virtual-to-physical mapping of the memory and highly accurate clock cycle timers that will enable him to prime and probe the cache with ease and deploy the attack. On the other hand, the user-space attack has no access to neither of the aforementioned functionalities, so the researchers have proposed and implemented two replacements that although not as accurate, can be used to carry out successful unprivileged attacks.

**Figure 14:**
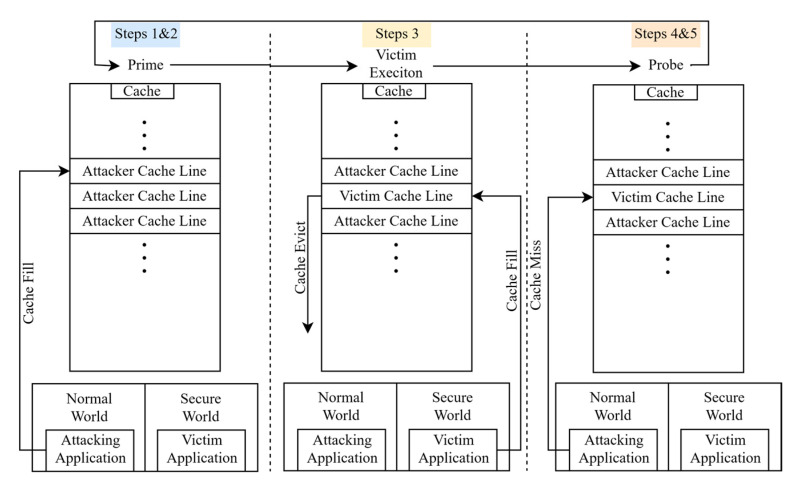
Prime + probe process of TruSpy.

Both attacks are implemented as prime + probe cache attacks since it does not require memory sharing between the attacking and the victim process. This attack consists of five steps, first the attacker finds what memory addresses should be used for the priming of the cache by working out the mapping from the virtual address space to cache sets. In the second step the attacker fills the cache with its own memory so as to force any future cache access attempts from the victim process to fail. This step is the prime phase that will provide the attacker with a known state of the cache before he hands the execution to the victim process. Moving forward, the victim process from the trusted world takes over and changes the state of the cache during its execution which cannot be interrupted due to the fact that it resides in the trusted world. During the fourth step, the attacking process takes back control and measures the difference between the known cache state and the new cache state after the execution of the victim process. The attacker then stores the difference and proceeds to the second step again. The steps two to four are followed in a loop until enough data are recorded. In the last step the collected data are analyzed to recover any targeted secret information from the trusted world. The entire process is depicted in Figure 14.

#### 3.3.2. Other Side-Channel Attacks

The main problem with side channel attacks is their temporal and spatial resolution which are terms that define the granularity of the attack, that is, how detailed are the data extracted from the attack. These resolutions practically define the performance and the capability of an attack to extract the data required for the reconstruction of the targeted secret. Cachegrab [106] is a tool specifically developed for optimizing side-channel attacks against TEEs. This tool is designed to be analogous to an oscilloscope. It is capable of collecting trace data on multiple cache structures per execution, similar to the probes on an oscilloscope being connected to multiple contacts on a target board. With this tool, researchers from nccgroup (production company of Cachegrab) where able to recover 224 and 256-bit ECDSA keys from a Nexus 5X device. [107] In this attack they utilized not only the shared cache but also branch predictors to leak information about the nonce used in the ECDSA encryption procedure that where enough to completely recover the private key used.

Additionally, in [107] the researchers attacked implementations of AES-256 and AES-256 GCM that were found within the TrustZone TEE of a Samsung Galaxy S6. The attack followed the prime + probe method and through the usage of GPU parallelization in the analysis phase, the keys were recovered in a matter of minutes. (8 min for AES-256 and 70 min for AES-256 GCM)

#### 3.3.3. Fault Injection Attacks

##### Rowhammer on TrustZone (Electromagnetic)

**Figure 15:**
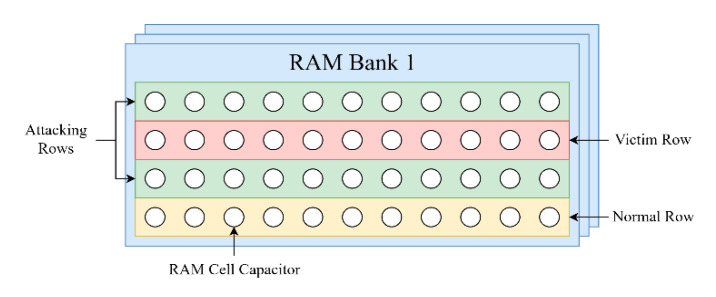
RowHammer attack.

In contrast with the previous side channel attacks that were based on the cache, the rowhammer attack exploits the physical design of the RAM. This attack [108] aims to induce errors to RAM entries within its capacitors by utilizing the fact that if many access and write attempts are made to neighboring RAM capacitors, then the contents of the target capacitor row can be altered. Like the name of the attack indicates, this attack cannot be applied on a per RAM capacitor level, but on a RAM row level with each row being composed of multiple capacitors. The attack is presented in Figure 15, where each RAM module is composed of multiple RAM Banks with each one of them containing multiple rows of RAM cell capacitors. The attacking rows are activated (and thus discharged) very frequently in order for this attack to work, the discharging of the capacitors in the attacking rows may cause the capacitors of the victim row to also discharge faster than normal. This will ultimately lead to memory corruptions in the victim row within the RAM, something that can affect RAM rows that contain TrustZone data, even if they are protected by the NS bit mechanism.

The attacker had to find a way to pinpoint the exact rows that were next to the desired victim rows. He developed a program that accessed the RAM row by row and timed each access attempt. Depending on this time he could deduce and map the entire RAM in its banks and rows. The final step to overcome was to bypass the cache usage that would cripple his attack as it would reduce the number of RAM activations frequency. This was trivially bypassed by adding to his assumptions the ability to execute code in kernel privileges that would disable the cache usage.

The attacker targeted the RSA CRT implementation, which is susceptible to the Bellcore attack, that existed in the TrustZone portion of the system. This attack is a differential fault analysis attack that requires only one faulty signature to be entirely broken in a CRT implementation, as long as no DFA countermeasures are in use. The attacker proceeded to monitor the execution of the RSA CRT as it signed content and when the timing was right, he began the attack. To that end, he developed a kernel module to specify the attacking rows that he used in order to attack rows adjacent to the sensitive memory rows. After he successfully corrupted the signature, it was easy to proceed to the next steps of the Bellcore attack that ultimately fully exposed the private key of the RSA.

##### CLKscrew (Voltage and Frequency)

CLKscrew [10] is a fault attack that is based on a modern device feature that allows software components to control CPU frequency and voltage for power management. By pushing the CPU to its operational limits, faults are induced in the computations of sensitive operations and through that, an attacker could obtain vital information for deducing sensitive data used in these operations. This attack can be done remotely, since it is software based and there is no need for physical access to the device. With a malicious kernel driver, a low privileged application can induce CLKscrew and extract cryptographic keys from inside the TrustZone TEE and can also manipulate the TrustZone trusted application validation into accepting self-signed code.

In this particular demonstration, the Dynamic Voltage and Frequency Scaling (DVFS) power management system is used to manipulate both the operating voltages and frequencies of each thread of the CPU. With the use of the DVFS the CPU can be overclocked and simultaneously undervolted in order to pass the fault inducing borders of the CPU. The attack is possible due to the fact that there are no safeguards to protect the CPU from operating in faulty frequency-voltage combinations and that hardware regulators operate across the TEE separation, allowing the attack to take place even during the execution of the trusted world. With faulty behavior frequency-voltage combinations identified the attacker needs to be assured that the attack will not self-fault the malicious code or any other non-targeted code. For that a custom kernel driver is used in order to bind the victim thread to a specific core and all the other applications to the rest of the cores, removing thus the threat of collateral damage during the attack. The kernel driver also disables interrupts during the fault injection in order to avoid any context switch that could happen.

**Figure 16:**
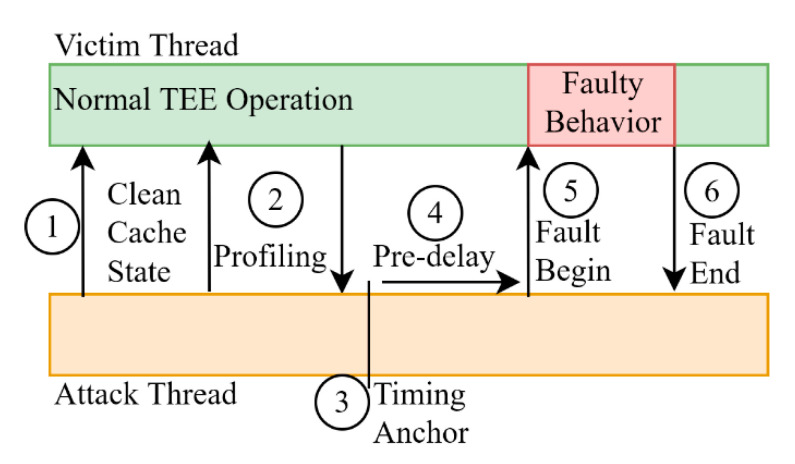
The CLKscrew attack.

The attack preparation begins by clearing any cache residue since in the next steps there will be use of a cache-based profiling to identify when the victim execution begins (step 1). In the next step, the attacker profiles the execution of the victim thread to identify a consistent point of execution just before the target code to be faulted, this point is named “Timing Anchor” (steps 2, 3). In some attack scenarios the timing anchor is not accurate enough and there will be a need to better fine tune the exact attack timing; this is implemented by trapping the attacking thread in a no-op loop until a predefined amount of time has passed by which the attack will start (step 4). During the attack, the frequency of the victim core will be raised to a specific value for a specific amount of time and then restore it to normal operating conditions (steps 5, 6) as shown in in Figure 16.

The attacker using this technique has managed to attack an AES implementation that he run in the trusted world. The application was a simple decryption tool that took as input encrypted messages and replied with the decrypted plain text given a stored secret key. By sending encrypted text to the application and inducing faults during specific rounds of the AES decryption phase he managed to infer the AES secret key by using a differential fault attack technique. The researchers have also been able to bypass the firmware verification mechanism of the device secure boot mechanism. This mechanism is an RSA based authentication that checked a signature on the hash of the firmware to be loaded with a hardware bound secret key stored in the trusted world. The researchers managed to fault the signature decryption process in order to force it to produce a hash that is equal to the hash of their own firmware so that the verification mechanism will accept this new firmware as a component signed by a trusted entity.

In a similar manner, VoltJockey [79], in an effort to additionally cover the tracks of a DVFS based attack, generates faulty behavior by just manipulating the voltage of the processor. This provides an increased difficulty due to the enforced restrictions which will undoubtedly create fewer effective faults than CLKscrew, but the resulting attack will be stealthier since the defender will not be able to detect the abnormal behavior by monitoring the frequency of the CPU.

##### BADFET (Electromagnetic)

Like the CLKscrew, BADFET [76] is a fault attack, that employs electromagnetic radiation to induce faults. Performing Electromagnetic Fault Injection (EMFI) attacks [109,110] is considered more challenging nowadays, due to the increase in CPU speeds and decrease of the component size in modern systems. BADFET proposes a solution, by defining the second order EMFI attacks, that targets a new attack surface for the EMFI based attacks. Instead of targeting the processor during sensitive operations, a second order EMFI attack, can target any component that the processor utilizes during the target operation (the memory, the system bus, various controllers etc.). With this technique the spatial and temporal resolution requirements are effectively reduced since the targeted components are less dense and they store exploitable information for more time.

The attack described in the paper, consists of two phases, in the first stage the BADFET platform is used to apply electromagnetic radiation on the RAM of the system (Cisco 8861 VoIP phone) during the boot in a specific time. A microcontroller is used to fine-tune the timing and the position of the emitter to increase the effectiveness of the attack. The faults induced in the memory, triggers a condition that exposes the debug CLI of the uBoot to the attackers. In the second stage, with the CLI available, a buffer overflow based trusted world vulnerability is found that allowed the researchers to attain read, write and execution capabilities in the secure world. Finally, the researchers have obtained a new CLI that run entirely in the secure world. This vulnerability exists due to the lack of user input validation in the TEE and the exposure of a function that can trigger a world change in the uBoot CLI.

### 3.4. Attacks Overview and Comparison

Based on the presentation of each attack we created a comparative table that aims at creating a synoptic view of each attack and what were the consequences with regards to the GlobalPlatform’s Protection Profile objectives (as analyzed in Section 2.2). More specifically, in the first column of Table 2 there is the name of each attack presented previously. In the next five columns we defined basic primitives that the attacker achieved; namely: (i) read for when trusted world memory reading was achieved, (ii) write for when trusted world memory write was achieved, (iii) execute for when execution in the context of a trusted application was achieved, (iv) kernel execute for when execution in the context of the trusted world kernel and (v) information extraction for when actual sensitive information was pulled from the trusted world. Depending on whether each primitive was achieved in each attack we use a black-filled circle to indicate that the primitive was achieved, a half-filled circle to indicate that the primitive can be trivially achieved but was not formally documented on the report and a white circle to show that the primitive was not achieved and to the best of our knowledge is hard to attain. In the final column, we specify the GlobalPlatrform protection profile objectives violated by each attack.

The most common primitives achieved in the documented attacks are the read primitive (21 achieved–3 possible), the write primitive (14 achieved–2 possible) and the information extraction primitive (12 achieved–6 possible). This is an expected outcome given the fact that reading and writing to memory is one of the most common initial pivots for attacks and a relatively easier to achieve primitive; as for information extraction it is a logic next step from the read primitive with side channel and fault injection attacks being the most common attacks that achieved this primitive. The least achieved primitives are the execute primitive (8 achieved–5 possible) and kernel execute primitive (7 achieved–4 possible).

## 4. Discussion and Observations

A TrustZone TEE is a high-value target. It has a privileged standing point in the context of access, due to its position in the architecture. It can conceal its state and data from other non-privileged modes of the platform -normal world- and also hosts a number of important services, whose security modification or compromise would be considered crucial from an attacker’s perspective. For the above reasons, a TrustZone TEE makes for a high-value target for someone with the required knowledge to exploit the weaknesses that exist in current implementations.

Generally, an attacker with the ability to execute code in the context of the secure world can fully compromise the system in various ways depending on the privileges he has, while also have access to secrets stored within the secure world. As a normal trusted application, he can access any resource that the trusted application might have access to:

- By using the secure world’s ability to map and write to all physical memory belonging to the normal world, an attacker can modify the kernel of the normal OS, thus infecting it even if no vulnerability is present in the normal world kernel.
- As the attacker has trusted application privileges, it can uncover any private data that it may hold in its memory. Things like loaded keys, fingerprint data and other sensitive information could be extracted, depending on which application is compromised.

Furthermore, an attacker that has kernel rights within TrustZone can manipulate resources from other trusted applications as well as the integrity of the kernel and other security parameters of the trusted world:

- Being able to access the secure file system available only to Secure World (SFS), he can extract cryptographic keys and other important data like fingerprints that the normal world is not privileged to access.
- The attacker’s ability to modify secure world memory gives him also the ability to compromise the integrity of every Trusted application.
- Lastly, the potential to modify qFuses, the one-time-programmable (OTP) elements that are used to enable and disable security and debug features is provided, thus bypassing the secure boot sequence, or enabling other capabilities that the vendor has chosen to disable on the platform.

**Figure 17:**
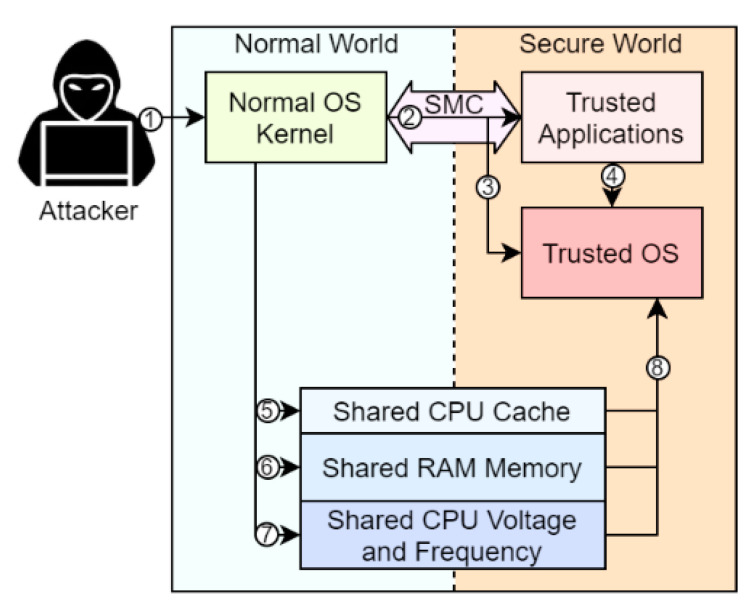
TrustZone attack paths.

Overall, the attack paths that an attacker could follow are depicted in Figure 17. Every attack scenario begins from the normal world (1) which usually requires that the attacker has kernel privileges in the OS. From there, the next step is either the SMC communication channel (2–4) or the shared architectural components of the system (5–8). In the first case, the attacker takes advantage of the escalated privileges and issues direct SMC calls that allow him to probe the various trusted applications or the trusted OS directly so as to discover any software exploitable bugs within the trusted code base. On the other case, the shared CPU cache (5) or the shared RAM memory (6) or the shared CPU voltage and frequency settings (7) are used to induce faults in secure TEE operations and to ultimately expose information from the secure world through side channel analysis (8).

### 4.1. SMC Exploitation

Most of the attacks against the TEE have a basic starting point in common; the communication system between the two worlds. This system provides the largest attack surface since the secure monitor calls (SMCs) provide a way for the user to pass potentially malicious input to the TEE. Since there is no default message authentication mechanism in TrustZone, an attacker with kernel privileges, can issue any custom SMC and perform fuzzing and man in the middle attacks in order to discover flaws in the trusted world and exploit them.

The communication starts from the normal world application that places arguments in a shared memory or some specific registers and calls the TEE kernel driver to initialize a world change. Then, the normal world kernel sends the required SMC which will make the secure monitor give control to the TEE. In a normal case, the SMC is issued by a kernel driver that will also authenticate the calling application, although if an attacker gains kernel privileges he can issue his own SMCs utterly bypassing this authentication. After that, the TEE processes the arguments provided and calls the trusted application to perform the actions specified. Since the input is controlled by the attacker it can contain crafted arguments that will be passed to the TEE, which has very limited mechanisms to verify the validity of the message (at least in current implementations) aside from sanity checks that many times fail to catch some malicious inputs. The TEE has no other option than to act blindly, using the information provided in the message. Some might argue that there is a way to verify the calling application by using the universally unique identifier (UUID), but the UUID is part of the SMC, and as such, it can be replicated and consequently be nullified as a security measure.

Another crucial aspect that arises from the above cases is that when an OEM/platform designer extends a TEE with more services, the implementation of input validation mechanisms on these added SMC calls are not always coherent and of the same quality as that of the initial mechanisms designed by the TEE vendor [83]. From the above-published research, we can see that the OEM/platform vendor can either copy the TEE designer model of input sanitization or design their own validation checks on the implemented function calls of the added Trusted applications. This second option has the drawback that the chosen mechanisms may not address all scenarios of bad input, because of the non-expertise of the developer in security design, expanding probabilistically an attacker’s ability to execute arbitrary code in the context of the TEE, as can be seen from the presented attacks.

Another issue that arises from the fact that manufacturers extend the secure world with new functionality is the dramatic increase in trusted code base. With the ever-increasing need for secure applications, it is hard to strike a good balance between what part of an application is implemented in the normal world and what in the secure world. This has led to an expansion of the secure world since developers choose to implement more parts of the application in the secure world, a fact that exposes it to greater danger since the newly introduced code might contain new and unforeseen vulnerabilities which could be exploited through the SMC mechanism.

This problem has been identified in [111] where the researchers have identified the lack of a protection mechanism of the TrustZone communication system which could lead to man-in-the middle, fuzzing and denial of service attacks. They proposed a scheme which aims to alleviate this issue by restricting TrustZone access through access control lists and by encrypting and authenticating the communication with session keys.

Concluding, a basic design flaw of the trusted execution environment is its communication system, although there can be some security measures within the normal world, an attacker with root privileges can easily bypass them. There are some attacks that do not employ this communication channel, such as the aforementioned side channel and fault attacks, but the most widely documented method of attacking the TEE is through this communication channel using malicious crafted SMCs.

Finally, it should be considered that SMC calls are not the only mechanism with the ability to switch world context, thus not the only probable way of exploitation. Interrupt handling and bus signals from the corresponding controllers, shared memory interfaces found on some designs and data parsing of intentionally corrupt disk content from eMMC blocks are other possible avenues of exploitation, although no research has been published indicating vulnerabilities through the above mechanisms yet.

### 4.2. Protection Mechanisms of Trusted OS

Something to consider also is the impact and importance of GlobalPlatform’s standardization that most of the current implementations of TEEs are compliant with. According to the official definition from GlobalPlatform: “The TEE is an execution environment that runs alongside but isolated from the device main operating system. It protects its assets against general software attacks. It can be implemented using multiple technologies, and its level of security varies accordingly.” [26] Comparing the official definition with the current situation we can deduce that problems in one of the crucial requirements exist. The ability of TEEs to withstand attacks from exposing an interface to the normal OS, which can be considered the most standard/general and the most exposed interface, is defective. Furthermore, most TEE implementations lack basic internal security mechanisms such as ASLRs and stack cookies [1,29].

Modern rich OS include a set of security mechanisms that increases the difficulty of exploiting a software vulnerability. Furthermore, due to the closed nature of TEE OS, there is no public information regarding the support of security mechanisms. A recent article in [9] discovered that the two major TEE implementations (Qualcomm and Kinibi) have a limited number of security features implemented. In particular, Kinibi offers no form of ASLR and all trusted applications are loaded into a fixed address with Qualcomm TEEs offering only a weak ASLR implementation. That is, the amount of entropy offered by QSEE’s ASLR implementation is limited by the size of the contiguous range of memory where trusted applications are loaded. According to [9], the ASLR enabled by QSEE is limited approximately to 9 bits, which means that with 355 guesses, an attacker would have a 50% chance of correctly guessing the base address of a trusted application image. Another security mechanism called stack cookie, which prevents stack overflows has been implemented in QSEE but not in Trustonic’s implementation. Finally, both QSEE and Trustonic do no support guard pages (i.e., a security mechanism to separate various memory segments and block overflows from one segment reaching to another segment).

A deduction from the above presented cases is that—at least on specific implementations as QSEE- the security boundary between Trusted applications and the TrustZone kernel is weak [83]. When the attacker has managed to break through to the secure world and has taken command of a Trusted application, the intermediate communication mechanism between the Trusted applications and the TrustZone kernel is designed such a way that no input validation takes place, thus allowing the attacker to compromise the kernel and other Trusted applications with ease.

When this weak security implementation is combined with the increasing number of trusted applications, it is apparent that the security of each one of these applications is compromised in an exploitation incident. This way, a need for a more effective solution arises in order to maintain the security of each trusted application. In [91,112], the researchers have identified this issue and they proposed a TEE virtualization (TEEv) architecture that supports multiple isolated and restricted TEE instances running under the same CPU with a minimal hypervisor. TEEv allows different TEE implementations to run in isolation on the same smartphone and to host their own set of trusted applications. Coincidentally, a compromised TEE in this architecture cannot affect its peers or the normal world. There are many similar virtualization research solutions such as [113], PrOS [114], TrustICE [115], vTZ [116] and Sacntuary [117].

### 4.3. Shared Resources

A crucial design flaw of all TEEs is the sharing of resources. TEEs were designed to provide an ad hoc security mechanism that required very little hardware adjustments to work without the need of extra hardware to be installed on the device. That is the reason why the CPU cores, the CPU caches, the storage, and the RAM of the device are all resources that are shared between the secure and the non-secure worlds. There are strong security mechanisms that protect the resources of each world such as the NS bit that taints each message and memory entry as secure or non-secure. With the support of mechanisms that enforce the NS bit policy, unauthorized entities cannot access sensitive resources, but although this architecture might seem sound, it does not account for side channel attacks.

As we have seen, the fact that these resources are shared between the two worlds has led to serious side channel vulnerabilities. These attacks range from basic sender and receiver side channels that can be used to secretly transfer data from one world to another, all the way to spying attacks that can steal sensitive data from trusted applications during their execution. That is why manufacturers should either reserve dedicated hardware for the TEE, something that will increase the production costs, or implement mechanisms that scramble the data that the side channel attacks collect [118,119], rendering them unusable. The latter method is the least expensive, but it includes the risk that it might not catch all attack cases, in contrast, the first method is expensive (due to the need for extra hardware) but it provides assurances that the data are safe from shared resource side channel attacks.

Furthermore, just separating the hardware resources is not enough, as there are other side channel attacks that are not based on this architectural design. These side channel attacks are based on external physical measurements or manipulations of the device that the manufacturers cannot shield against. In this case, the only possible solution is through the software, that should be hardened in order to not leak sensitive data. More specifically, the software should be optimized to have fixed power consumption and runtime execution regardless of the configuration of the targeted application. This will be able to protect against passive side channel attacks that only measure these specific characteristics of each execution. Finally, the software should also be hardened against hardware fault injections that could lead to exposing secret data.

### 4.4. Other Issues

Another very important aspect we should consider is the closed source nature of TEE implementations. Most designs are not public, resulting in an architecture that is not analyzed in an open manner for security defects. This closed box design and the non-disclosure agreement policies that follow it, hinders the ability of security researchers to investigate the products for existing vulnerabilities [120]. Only recently has Qualcomm released the specifications for the Qualcomm TEE secure boot procedure and trusted application image authentication.

A TEE should provide assurance based on its secure design (verified microkernel, isolation between tasks, secure input validation, overflow protection etc.) and by hardware mechanisms that provide the ability to isolate its state from “external” sources, like code running in the REE. From the presented cases we can see that the design of the most widespread implementation in not optimally secured, allowing for exploitation primitives that can totally break the security design.

### 4.5. Solutions and Future Work

Given the severe security issues we have identified and analyzed, here we propose a set of possible solutions and improvements that could be adopted in TEE implementations so as to increase their security and ultimately mitigate to the highest possible degree the vulnerabilities we presented. Once again, we follow our vulnerability categorization to highlight the solutions that we propose for each type of vulnerability refereeing to Figure 17 when required.

#### 4.5.1. Solutions for Software Vulnerabilities

When it comes to software attacks, the main identified issues are entirely based on basic software attacks (control flow graph manipulation through buffer overflows, malformed input etc.). Developers of trusted applications should be careful to maintain secure and bug-free code that does exactly what it is supposed to do, this can be achieved by keeping the trusted code base at the minimum, effectively reducing the chance that a bug will exist and making the debugging process easier. The solution here is identical to common software security development procedures that includes (i) integration of secure software development lifecycle functions such as static and dynamic code analysis, fuzzing etc. and (ii) open sourcing security critical software so as to allow close scrutiny of the source code from a wider range of experts.

Furthermore, as identified in [9], common TrustZone-based TEEs lack common OS-level security techniques (ASLR, stack canaries etc.) which allows software exploitation to manifest in vulnerable binaries, something that can be avoided if TEE implementors add these techniques to their solutions. In addition, drivers and software proxies that communicate with the TEE must properly sanitize all requests, authenticate, and verify the senders of these requests. These entities are the gatekeepers to the secure world and the first line of defense against attackers, that is why TEE implementors should pay attention to secure these elements in order to mitigate the largest attack surface against the TEE. This of course needs further research so as to compare the computational overhead in comparison to the additional security provided. The aforementioned solutions aim to fortify the “Trusted Application” and the (4) attack path of Figure 17.

#### 4.5.2. Solution for Architectural Vulnerabilities

The architectural vulnerabilities stem from the building block concepts of common TrustZone TEE implementation with a central part being the SMC communication channel witch attackers use to gain probing capabilities to the internals of the TEE that allows them to gain an initial foothold within the secure world. As proposed in [113], an authenticated and encrypted channel will provide an additional layer of security to the SMC communications and prevent arbitrary SMC usage from kernel-privileged users. This of course only locks the door with better security and does not solve the software vulnerabilities that the attackers use to exploit the trusted applications (see Solutions for software vulnerabilities). On the other hand, there is a wide array of other architectural vulnerabilities (such as the downgrade attack) which are rooted in OS-level logic problems and unused TEE features. These issues can only be solved through more detailed and coherent implementation and usage of the TEEs in terms of security and bug searching. Once again, open sourcing parts of or entire TEE implementations will allow the code base to be thoroughly checked for issues by a wider array of experts. These solutions aim to secure the “Trusted OS” as well as the (2), (3) and (4) attack paths of Figure 17.

#### 4.5.3. Solution for Side Channel Vulnerabilities

Side channel attacks are enabled by the fact that the secure and normal world share common resources (CPU, cache, and RAM) which allow attackers to inject faults while also measuring execution times, power consumed and cache/RAM usage. As we have seen (Section 4.3) this core issue can only be solved by either adopting side channel resistant cryptographic algorithms (with constant execution times and constant power consumption) or changing the state of the shared resources by separating them (as in different hardware chips) or by changing how they work by introducing random noise that can halt these attacks [121,122]. Choosing the optimal solution is a research topic that we propose and will require the cost/benefit analysis of adding new hardware to a SoC or having higher overhead side-channel-resistant algorithms. Furthermore, detection of side channel attack attempts is possible by orchestrating the shared CPU performance counters which are able to identify the number of cache-misses; a number that will spike during the attack. With this detection mechanism, the execution can stop when attacks are identified and thwart the attempt [123]. These solutions will block the attack paths (5–8) of Figure 17.

Finally, a more generic guideline is that all TEE implementations should be made open to be publicly evaluated and improved. As it is widely recognized, security systems should be secure by design and not through the obscurity of their implementation. ARM TrustZone is no exception and the industry should avoid relying on the secrecy of the design or implementation as the main method of providing security.

### 4.6. Future Directions

In this section we present the identified research directions. More specifically, in light of our analysis, we believe that the future work in this area should deal with one or more of the following research issues:

- Currently there is no formal threat model for the TrustZone TEE. This kind of model would greatly benefit the development and security research of future TEEs as it will set the focus on the most important security threats that a TEE can be affected by.
- More tools are required to evaluate TEE implementations and discover security bugs. For example, custom fuzzers [42] (model based or coverage guided) and static analysis tools aware of TEE protocols that will be able to adapt to the unique requirements of secure-normal world communication. Another possible method is the use of symbolic execution for trusted applications such as [124] or dynamic analysis such as [125].
- As Cortex-M architecture has been optimized for low-power devices such as IoT, the underlying mechanisms of TrustZone technology for Cortex-M and Cortex-A processors are different. Therefore, research efforts must focus also on TEE implementations built for TrustZone Cortex-M variant such as [126].
- Development and widespread availability of TrustZone based hardware platforms is a crucial parameter (for both Cortex-A and Cortex-M) that will allow researchers to evaluate various TEE implementations.
- Security is not the only aspect of TrustZone which requires attention. More works such as [127] are required to investigate TrustZone performance-wise and energy-wise.
- Research efforts should focus on reducing the TCB size of the TEE OS and the optimal distribution of functionalities between the trusted and normal world applications.
- The security of the communication between various parts of a heterogeneous SoC is crucial. That is, novel solutions for the authentication and security of the communication channel between the secure and normal world should be investigated.
- A common use of TrustZone technology is data security such as DRM. However, the powerful capabilities of TEE technology can be leveraged to enable many more interesting privacy-enhancing solutions. Notable cases are the development of Private Contact Tracing (PCT) (e.g., to track COVID-19 contacts) [128] and privacy preserving machine learning at the level of devices [129]. More work is required to unleash the potential of TrustZone and showcase more interesting application scenarios.
- Further investigations for underlying TEE vulnerabilities that are not based on trusted application vulnerabilities but on architectural issues of TEEs could shed light on how the TEE isolated environment could be better secured and also showcase its limitations. An example of such a research [130] implements vulnerable trusted applications that are exploited through memory corruptions on the OP-TEE platform.

## 5. Conclusions

From 2013 and on, research has surfaced that proves that the current implementations of ARM TrustZone implementations are not immune from attacks. The aim of this paper was to provide an analytical and educational analysis of TrustZone-based TEE vulnerabilities and related attacks. To this end, we created a taxonomy that divided them into three generic categories: (a) software, (b) architectural and (c) hardware attacks. We elaborated on each attack category focusing on subtle details that are important for the understanding of these attacks. Some of the presented exploits were highly complex and required skillful attackers (such as extraction of Master keys), while other ones (such as the downgrade attack) were rather trivial. Regardless of their complexity, all presented attacks are real-world with practical impact on the security of the underlying device. We conducted a root cause analysis of the said vulnerabilities to discover the underlying causes and pinpoint the nature of the security flaws. Our conjecture is that TrustZone based TEEs can be considered as still immature due to the previous evidence. However, they are a step to the right direction, with the TrustZone technology providing all the tools needed to develop a secure environment for next generation sensor and IoT networks. Most critically, this work showed that a more open and mature approach must be evaluated, with a more detailed attention to their design from the perspective of security.

## Figures and Tables

**Table 1:** Kinibi vulnerabilities of the unbox your phone attack reported as SVEs.

Vulnerability	Description
**Vuln 0**: SVE-2017–8888	Authentication Bypass in the tlc_server binary.
**Vuln 1:** SVE-2017–8889	Buffer Overflow in the TLV storing struct within the ESECOMM trusted application.
**Vuln 2:** SVE-2017–8890	Buffer Over-Read of the TLV containing buffer sent to the ESECOMM trusted application.
**Vuln 3:** SVE-2017–8891	Buffer Overflow in parse_ca_cert() of a 32-bit buffer with a 400-bit input.
**Vuln 4:** SVE-2017–8892	Buffer Overflow in the parsing of a Diffie-Hellman parameter in the parse_scp_param() function.
**Vuln 5:** SVE-2017–8893	Buffer Overflow of the “envelop_len” of the TCI shared buffer.

**Table 2:** A comparison of the analyzed attacks that showcases the methodology used, the primitives achieved, and the GlobalPlatform Protection Profile objectives violated by each attack. The Kernel Normal World (KNW) mark shows that the primitive targets the kernel of the normal world. (○ Not achieved ◒ Not achieved but trivial ● Achieved).

Attack	Method	Read	Write	Execute	Kernel Execute	Info Extraction	GlobalPlatform Protection Profile Objectives Violated
Bits Please 1 [28,82]	Logic Exploitation	○	●	●	○	○	O.OPERATION O.RUNTIME_INTEGRITY O.TA_ISOLATION
Bits Please 2 [71,83]	Logic Exploitation–Return Oriented Programming	○	●	●	●	◒	O.OPERATION O.RUNTIME_INTEGRITY O.TA_ISOLATION O.TEE_ISOLATION
Bits Please 3 [84]	Logic Exploitation	●	●	●	●	●	O.OPERATION O.RUNTIME_INTEGRITY O.TA_ISOLATION O.TEE_ISOLATION O.TYO.TEE_D O.TEE_DATA_PROTECTION O.TRUSTED_STORAGE
Unbox Your Phone [11,85,86]	Logic Exploitation & Buffer overflow/overread	●	●	●	●	◒	O.OPERATION O.RUNTIME_INTEGRITY O.TA_ISOLATION O.TEE_ISOLATION
Here Be Dragons [87]	Failed Memory Validation	●	●	●	●	○	O.OPERATION O.RUNTIME_INTEGRITY O.TA_ISOLATION O.TEE_ISOLATION
Reflections on trusting TrustZone [13]	Failed Memory Validation	●	●	●	●	○	O.OPERATION O.RUNTIME_INTEGRITY O.TA_ISOLATION O.TEE_ISOLATION
Unlocking the Motorola Bootloader [88]	Buffer Overflow	●	●	○	○	○	O.OPERATION O.RUNTIME_INTEGRITY O.TA_ISOLATION O.TEE_ISOLATION
Bypassing Secure Boot [89]	Bad use of cryptography	●	●	◒	◒	◒	O.OPERATION O.INITIALISATION O.RUNTIME_INTEGRITY O.TEE_ISOLATION
TRUSTNONE [90]	Logic Exploitation	◒	●	◒	○	○	O.OPERATION O.RUNTIME_INTEGRITY
Attacking Your Trusted Core [29]	Logic Exploitation	●	●	●	●	○	O.OPERATION O.INITIALISATION O.RUNTIME_INTEGRITY O.TEE_ISOLATION
EL3 Tour [91]	Buffer Overflow	●	●	◒	◒	◒	O.OPERATION O.INITIALISATION O.RUNTIME_INTEGRITY O.TEE_ISOLATION
Unearthing the TrustedCore [92]	Bad Usage of Cryptography and Buffer Overflow	●	●	●	●	●	O.OPERATION O.RUNTIME_INTEGRITY O.TA_ISOLATION O.TEE_ISOLATION O.RUNTIME_CONFIDENTIALITY O.TEE_DATA_PROTECTION O.TRUSTED_STORAGE
Boomerang [14]	Failed Use of Memory Sanitization	● (KNW)	● (KNW)	○	○	○	O.OPERATION O.RUNTIME_INTEGRITY
Downgrade [95]	Failed Usage of Version Control of TAs	◒	◒	◒	◒	◒	O.OPERATION O.TA_AUTHENTICITY O.RUNTIME_INTEGRITY
FPGA SoC [96,97]	FPGA based manipulation of AXI interconnect	●	●	○	○	○	O.OPERATION O.RUNTIME_INTEGRITY
CoreSight [98,99]	Exploitation with ARM debugger	●	○	○	○	●	O.OPERATION O.RUNTIME_CONFIDENTIALITY O.TRUSTED_STORAGE
DeepSleep [100,101,102]	Failed validation of TZ image after device wake up	◒	◒	◒	◒	◒	O.OPERATION O.INITIALISATION O.RUNTIME_INTEGRITY
Cache Timing Attack [75]	Timing of access attempts to the cache	●	○	○	○	●	O.OPERATION O.RUNTIME_CONFIDENTIALITY O.TRUSTED_STORAGE
Prime + Count [103]	Timing of access attempts to the cache with a known initial state	●	○	○	○	●	O.OPERATION O.RUNTIME_CONFIDENTIALITY O.TRUSTED_STORAGE
ARMageddon [12]	Flush + Reload, Flush + Flush, Evict + Reload cache timing attacks	●	○	○	○	●	O.OPERATION O.RUNTIME_CONFIDENTIALITY O.TRUSTED_STORAGE
TruSpy [73,105]	Prime + Probe cache timing information exfiltration	●	○	○	○	●	O.OPERATION O.RUNTIME_CONFIDENTIALITY O.TRUSTED_STORAGE
Cachegrab [106,107]	Optimized Side Channel Attack on TrustZone	●	○	○	○	●	O.OPERATION O.RUNTIME_CONFIDENTIALITY O.TRUSTED_STORAGE
Rowhammer [108]	RAM Fault Injection	●	○	○	○	●	O.OPERATION O.RUNTIME_CONFIDENTIALITY O.TRUSTED_STORAGE
CLKscrew [10]	Voltage & Frequency Based Fault Injection	●	○	○	○	●	O.OPERATION O.RUNTIME_CONFIDENTIALITY O.TRUSTED_STORAGE
VoltJockey [79]	Voltage Based Fault Injection	●	○	○	○	●	O.OPERATION O.RUNTIME_CONFIDENTIALITY O.TRUSTED_STORAGE
BADFET [76]	Electromagnetic Fault Injection	●	○	○	○	●	O.OPERATION O.RUNTIME_CONFIDENTIALITY O.TRUSTED_STORAGE

## Data Availability

Not Applicable.
